# Volcano-tectonic deformation in the Monti Sabatini Volcanic District at the gates of Rome (central Italy): evidence from new geochronologic constraints on the Tiber River MIS 5 terraces

**DOI:** 10.1038/s41598-019-47585-8

**Published:** 2019-08-08

**Authors:** F. Marra, F. Florindo, B. R. Jicha, S. Nomade, D. M. Palladino, A. Pereira, G. Sottili, C. Tolomei

**Affiliations:** 1 Istituto Nazionale di Geofisica e Vulcanologia, Via di Vigna Murata 605, 00143 Rome, Italy; 2 Department of Geoscience, University of Wisconsin-Madison, Madison, USA; 3 Laboratoire des Sciences du Climat et de l’Environnement, LSCE/IPSL, CEA-CNRS-UVSQ, Université Paris-Saclay, F-91191 Gif-sur-Yvette, France; 4 Dipartimento di Scienze della Terra, “Sapienza” Università di Roma, Piazzale Aldo Moro 5, 00185 Roma, Italy; 5 UMR 7194 HNHP MNHN-CNRS-UPVD, Département Homme et Environnement du MNHN,1 rue René Panhard, Paris, 75013 France; 6 Ecole française de Rome, Piazza Farnese, 00186 Roma, Italy

**Keywords:** Tectonics, Volcanology

## Abstract

The accumulation of magma within the Monti Sabatini Volcanic District (MSVD), Italy, coupled with the extensional tectonics of the region, pose both volcanic and tectonic hazards to the city of Rome, located 20 km to the southeast. We combine ^40^Ar/^39^Ar geochronology of volcanic deposits and a geomorphologic/stratigraphic/paleomagnetic study of fluvial terraces to determine the recurrence interval and the time elapsed since the last eruption of the MSVD. Moreover, we provide a date for the youngest known eruption of the MSVD and assess the timing of the most recent volcanic phase. Results of this study show: (i) The most recent eruptive phase occurred between 100 ka and 70 ka; (ii) the anomalously high elevation of the MIS 5 terrace indicates that it was concurrent with 50 m of uplift in the volcanic area; (iii) the time since the last eruption (70 ka) exceeds the average recurrence interval (39 ky) in the last 300 ky, as well as the longest previous dormancy (50 ky) in that time span. (iv) the current duration of dormancy is similar to the timespan separating the major explosive phase that occurred 590–450 ka.

## Introduction

**Figure 1:**
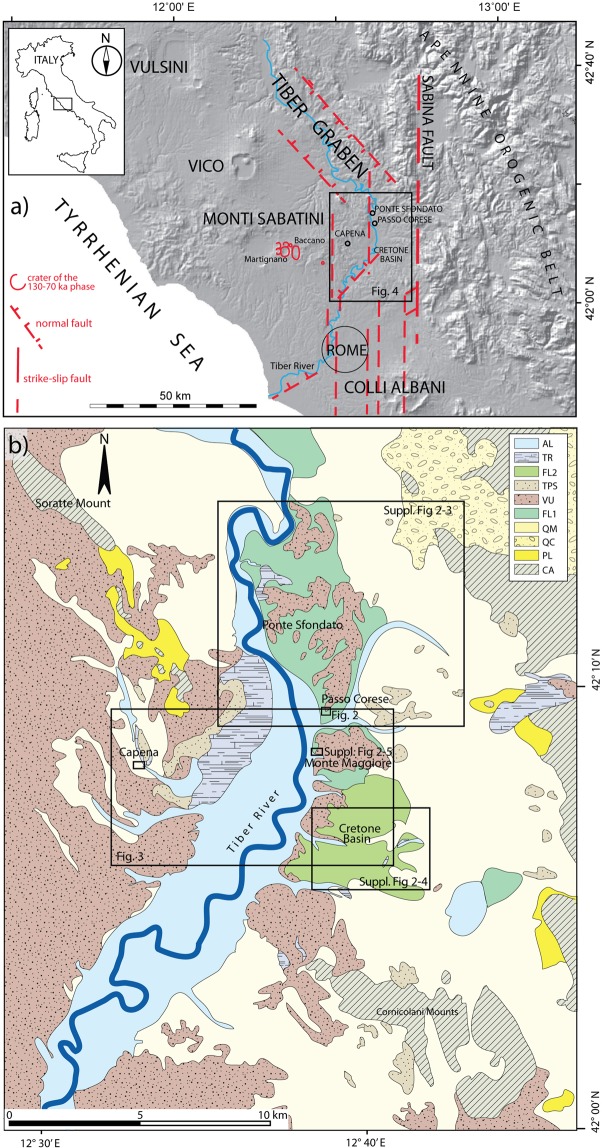
(**a**) Digital elevation map (DEM) of the studied region, showing the principal tectonic features, the volcanic districts of the Roman Province, and location of the detail maps (boxes) of the investigated sites. Modified after DEM TINITALY/01 square WA 6570, used with permission by the Istituto Nazionale di Geofisica e Vulcanologia, Rome, (**b**) Geologic setting of the study area. Location of the sectors investigated in the present study is shown. AL: Recent and modern alluvia (Holocene), TR: travertine (Upper Pleistocene), FL2: Fluvial-lacustrine deposits younger than 500 ka (previously mapped as “pedogenized bedded tuffs”), TPT: Pedogenized bedded tuffs with travertine intercalations, VU: Primary volcanic deposits, FL1: fluvial-lacustrine deposit older than 500 ka, QM: Marine to transitional deposits of Quaternary age (clay, sand and  conglomerate), PL: Marine clay deposits of Pliocene age, CA: Meso-Cenozoic carbonatic and silico-clastic deposits. Hand drawn based on authors re-interpretation of the Sheet 144 “Palombara Sabina”, 1:100.000 Geologic Map of Italy by Servizio Geologico Nazionale (http://193.206.192.231/carta_geologica_italia/tavoletta.php?foglio=144).

The magnitude and patterns of deformation of a volcanic field provide constraints on the magmatic processes operating beneath a volcano. A recent geomorphological study^1^ reconstructed a series of paleo-surfaces along a 40-km-long stretch of the Tiber River Valley north of Rome, between Magliano Sabina and Monterotondo, located at the eastern margin of the Monti Sabatini Volcanic District (MSVD) (Fig. 1a). These paleo-surfaces are interpreted as fluvial terraces formed through the interplay between regional uplift and glacio-eustasy (e.g.^2^) during a regressive phase that formed the hydrographic network of the Paleo-Tiber River since the end of the Santernian (1.78–1.5 Ma; lower Calabrian)^3^. The reconstructed rates of uplift during the last 1.8 Ma recognized two major pulses: 0.86 through 0.5 Ma, and 0.25 Ma through the present time^1^. The coincidence of the uplift pulses with the ages of the main volcanic phases^1^ are interpreted as mainly driven by uprising magma bodies from a metasomatized mantle source of the Roman Magmatic Province (e.g.^4–6^). This “magmatic” uplift overlaps a smaller isostatic component on the Tyrrhenian Sea Margin of central Italy.

In the present study, we further refine the chronostratigraphy of the paleo-surfaces in the area previously investigated, providing five new ^40^Ar/^39^Ar age determinations on volcanic products intercalated within the terraced deposits. Moreover, we perform a new, more detailed chronostratigraphic and paleomagnetic study of a clay section within an ca. 15-m-thick sedimentary succession at Passo Corese (Fig. 1), whose deposition pre-dated the onset of the large explosive activity of the volcanic districts of the Roman Province at around 600 ka^7–11^ and references therein. Combined with the stratigraphic investigations in the nearby area of Ponte Sfondato, these new chronostratigraphic data allow us to firmly constrain paleo-surface elevations for the entire suite of terraces correlated with Marine Isotope Stage (MIS) 15 through MIS 5 in this area, and allow a reconstruction the regional deformation. Because of the vast knowledge of the volcanic history of this region, we are able to deconvolve the uplift associated with glacio-eustasy from that of magmatic processes, which is often not possible. Finally, we provide new ^40^Ar/^39^Ar age determinations on volcanic products of the most recent activity at the MSVD, which is now constrained to 100–70 ka, with the aim to better assess the recurrence times of eruptions and the time elapsed since the last eruption. Combination of these data have fueled a detailed assessment of the tectonic-volcanic hazards in this region, including the city of Rome, which is in progress under a dedicated research project sponsored by the Istituto Nazionale di Geofisica e Vulcanologia, Italy.

## Geological Setting

Retreat of the subducting Adrian microplate, associated with African/Euro-Asian plate convergence, caused the NE migration of an arched fold and thrust belt (the Apennine mountain chain) and the opening of the Tyrrhenian Sea^12,13^. Progressive NE migration of the marine basins was controlled by principal NW-SE normal faults and by NE-SW transfer faults^14^, which also represent preferential pathways for magma to rise through the crust and form volcanic vents^15^. Volcano-tectonic processes led to acidic volcanism during the Pliocene^5,7^, and culminated in the Middle Pleistocene with potassium-rich magmatism^4,16^. Regional uplift since 800 ka has been mainly related to explosive volcanism of the Roman Magmatic Province^6,17^. A wide coastal plain eventually became a NW-SE stretching fluvial valley, following the development of a graben-like structure linked to the birth of the volcanic districts (i.e., Vulsini, Vico, Monti Sabatini, Colli Albani, Fig. 1a). Their eruptive products, mainly pyroclastic-flow and air-fall deposits, and their reworked equivalents, are intercalated within the sedimentary successions deposited by the Tiber River and its tributaries in fluvial and lacustrine environments during Middle and Upper Pleistocene (Fig. 1b). The geologic evolution of this area was therefore driven by the interplay among tectonics, volcanism, sedimentary processes, and glacio-eustacy^18^ and references therein.

## Results

### ^40^Ar/^39^Ar data

Eight new ^40^Ar/^39^Ar dates from primary as well as reworked volcanic deposits spanning 40 km of the Paleo-Tiber River range from 614 to 70 ka (Table 1). While dating of primary deposits provides the eruption age and the time of emplacement within the sedimentary successions, the youngest crystal population of reworked products provide constraints on the age of one of the eruptions sourcing the deposit. Full datasets are provided in Table S1–6 of Suppl. Material #1A. Comments and detailed description of petrographic/depositional features of the samples is provided in Suppl. Mat. #1B. Ages reported in Table 1 and within the text are calculated using the total K decay constant of^19^ and the flux-standard ACs-2 age of 1.1864 Ma^20^.

**Table 1:** Geochronology data - All ages calculated relative to 1.1864 Ma Alder Creek sanidine standard (Jicha *et al*.^20^).

Sample #	Material	Number of crystals			MSWD	Weighted mean age (ka) ± 2σ
^**40**^ **Ar/** ^**39**^ **Ar single crystal fusion data LSCE**						
CAP2	sanidine	11	of	11	0.95	141.8 ± 3.0
CAP3	sanidine	11	of	11	0.81	151.0 ± 2.4
OM-1	sanidine	4	of	14	1.06	403.0 ± 5.8
PC6	sanidine	8	of	10	1.17	591.3 ± 2.6
PC5	sanidine	5	of	11	1.13	614.3 ± 3.4
^**40**^ **Ar/** ^**39**^ **Ar single crystal fusion data WISCAR LABORATORY**						
BMU	sanidine	6	of	20	1.09	99.3 ± 2.7
MAR-3	sanidine	14	of	14	1.16	70.0 ± 3.3
ACQ	sanidine	5	of	17	0.71	82.5 ± 4.4

using the decay constants of Min *et al*.^19^.

### Paleomagnetic data

Magnetic susceptibility ranges in intensity from 12.2e-5 SI to 15.3e-05 SI with an average of 13.5e-05 SI. The NRM ranges in intensity from 2.7e-04 to 10.0e-04 A/m with an average of 6.0e-04 A/m. ChRM components were determined from principal component analysis using data from a minimum of three demagnetization steps. The magnetization is dominated by a single, normal polarity component (Suppl. Fig. #2-1b,c), isolated at peak fields ≤40 mT, proving that the sedimentary succession encompassing the investigated lower clay layer C2, the intermediate gravel layer G1, and the upper clay layer C1 was deposited after the Matuyama-Brunhes reversal (ca. 773.1 ka, end of MIS 19^21^). This is consistent with ^40^Ar/^39^Ar age of 614.3 ± 3.4 ka (Table 1) yielded by the tephra layer intercalated in the unconformably overlying sedimentary succession correlated with MIS 15 (Suppl. Fig. #2-1a). In addition, as evidenced by the efficiency of AF cleaning, the temperature dependence of low-field magnetic susceptibility (Suppl. Fig. #2-1d) indicate that magnetite is the dominant ferromagnetic mineral.

### Chronostratigraphy

Detailed stratigraphic description of the investigated sections is provided in Supplementary Material #2. Key chronostratigraphic evidence occurring in the investigated area is illustrated in the following paragraphs.

### Passo Corese

**Figure 2:**
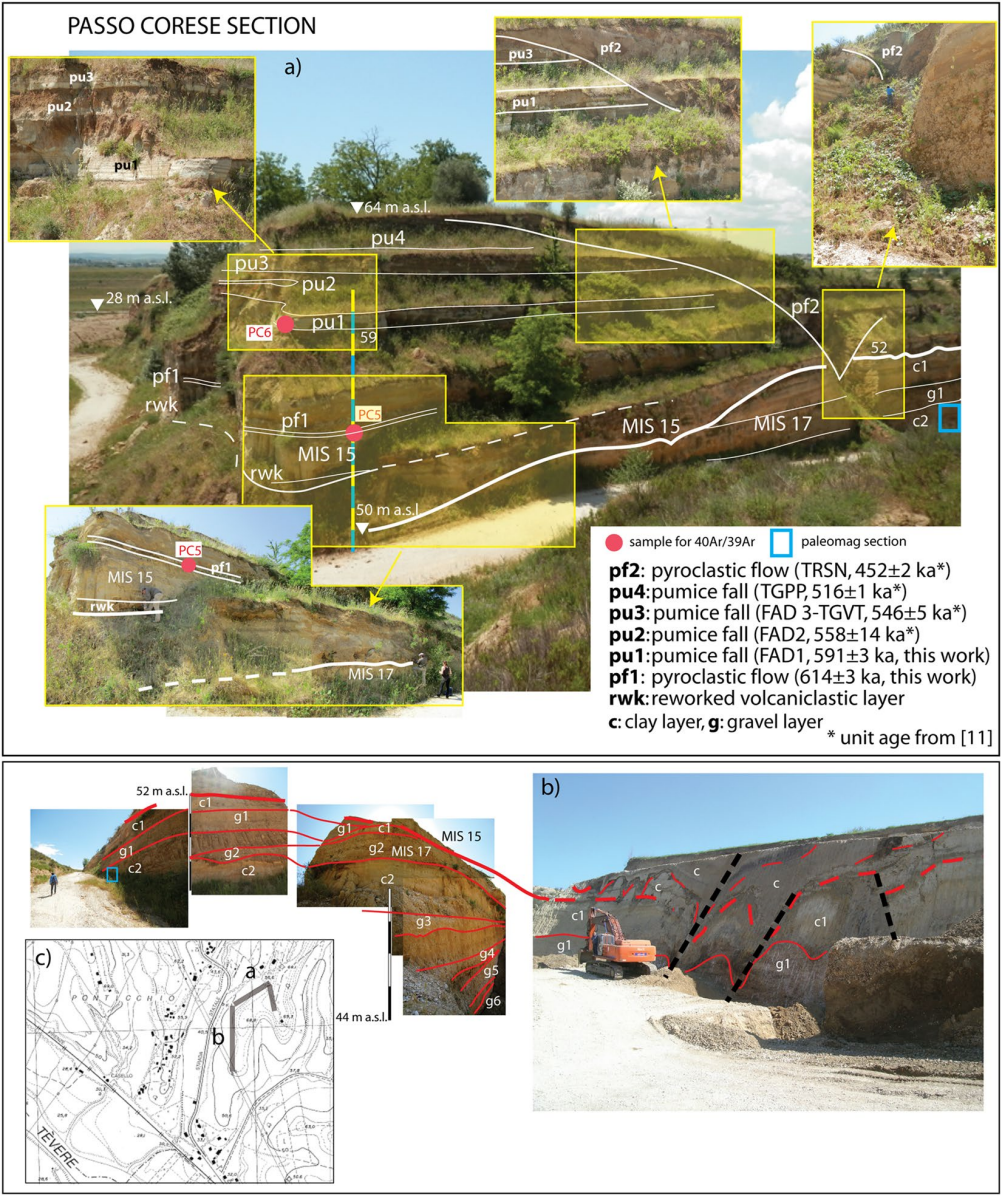
(**a**) Photographs by authors of northern face of Passo Corese quarry showing the outcropping sedimentary and volcanic deposits, and the sampling for ^40^Ar/^39^Ar dating and paleomagnetic investigations, (**b**) Authors’ photographs of eastern quarry face showing the intense tectonic deformation affecting the clay and gravel layers of MIS 17 and MIS 15 aggradational successions (see text for explanation); (**c**) location of the quarry (1:10.000 topographic base by Regione Lazio; http://dati.lazio.it/catalog/it/dataset/carta-tecnica-regionale-1991, available under Creative Commons Attribution License (https://creativecommons.org/licens/by/4.0/)).

Combined ^40^Ar/^39^Ar and tephrostratigraphic constraints on the uppermost sedimentary succession cropping out at Passo Corese (Fig. 2 and Suppl. Fig. #2-2a) support its correlation with the MIS 15 aggradational phase related to glacial termination VII. For the paleoclimatic implications of the methodological approach and the conceptual model of aggradational succession adopted in this paper, we address the readers to the previous literature (e.g.^22–25^).

Paleomagnetic constraints, indicating normal polarity (Suppl. Fig. #2-1b) for the clay section underlying the basal unconformity of the MIS 15 succession, provide correlation of the lower sedimentary succession with the previous aggradational phase of MIS 17 and glacial termination VIII, which post-dates the Matuyama-Brunhes magnetic reversal (Suppl. Fig. #2-2b).

The detailed chronostratigraphic framework described above and in Suppl. Mat. #2 allows us to constrain the elevation of the fluvial terrace of MIS 15 at ca. 59 m a.s.l. in this sector (Fig. 2 and Suppl. Fig. #2-2a), markedly lower than the elevation of 101–110 m a.s.l. estimated for the corresponding paleo-surface by^1^. This difference can be only subordinately attributed to the thickness of the overlying pyroclastic cover, whereas it is mainly due to tectonic dislocation, consistent with the fault displacement observed at Passo Corese, which affects the sedimentary deposits correlated with MIS 15 and MIS 17 (Fig. 2b). At this locality, a series of N-S to NE-SW striking fault segments are exposed along the south-western quarry face, which have repeatedly dislocated the sedimentary successions, as evidenced by several paleosoils affected by deformation, causing their dragging along the fault planes. These faults are sealed by the volcanic succession, indicating that prolonged syn-sedimentary tectonic activity spanned a time interval >700 ka to ~590 ka.

### Ponte Sfondato

Field survey at the nearby locality of Ponte Sfondato allowed us to map the geologic limit between the base of the volcanic succession and the underlying Pleistocene fluvial-lacustrine deposits in the geological map of Italy (Sheet 144 - Palombara Sabina, 1:100.000 Geologic Map of Italy, see Fig. 1b). The uppermost portion of the sedimentary succession is made up of a coarse gravel layer passing upwards to yellow sandy silt with freshwater gastropoda (Suppl. Fig. #2-3). The contact with the overlying volcanic deposits is not exposed but a series of nearby outcrops allowed us to recognize that it displays planar attitude in this area, and occurs between 85 and 90 m a.s.l. (Suppl. Fig. #2-3a). An almost complete exposure of the volcanic succession is observable in an ancient quarry cut at the top of the hilly area, between 110 and 90 m a.s.l. (Suppl. Mat. #2 and Suppl. Fig. #2-3b). The unexposed portion of the volcanic succession, with an estimated thickness of 3–5 m based on the nearby occurrence of the sedimentary succession at 85 m a.s.l., is correlated with that cropping out below the TGPP at Passo Corese, and spanning 591–546 ka (Suppl. Fig. #2-3b). Thus, the elevation of the fluvial terrace of MIS 15, is constrained to the top of the pre-volcanic sedimentary succession, at ca. 87 m a.s.l (Suppl. Fig. #2-3b), a value lower than the previous estimation of 101–110 m by^1^. These authors remarked that the top of the paleo-surfaces reconstructed through the geomorphological investigation did not reflect the actual elevation of the original alluvial plains, due to the deposition of a thick volcanic sequence since ca. 600 ka. As a consequence, the absolute elevation of each paleo-surface needs to be corrected by subtracting the thickness of the overlying volcanic deposits. On these grounds, the elevation of the MIS 15 fluvial terrace at Ponte Sfondato is assessed here at ca. 90 m a.s.l.

A comparison of the stratigraphy of the Ponte Sfondato and Passo Corese sections suggests the occurrence of ca. 30 m, post-sedimentary tectonic dislocation between the two sites (Suppl. Fig. #2-3b), thus accounting for the markedly lower elevation of the top of the MIS 15 aggradational succession and of the corresponding fluvial terrace at Passo Corese, with respect to the sectors unaffected by faulting.

### Cretone Basin

According to the detailed reconstruction of the Cretone lacustrine basin^26^, a suite of five main terraces are recognized in this sector that formed as a result of combined regional uplift, glacio-eustatic sea-level oscillations, and fault displacement. New ^40^Ar/^39^Ar data constrain the age of the lacustrine succession at Osteria Moricone, and refine the geomorphologic study of the Cretone Basin and the correlation of the terraced surfaces with the MISs.

A pyroclast-rich layer intercalated in the lacustrine deposits was sampled at Osteria Moricone (sample OM-1), along the eastern margin of the Cretone Basin, where the presence of fossil remains provides biochronologic constraints spanning MIS 15/13 (i.e.: *Hippopotamus antiquus*, *Axis eurygonos*) through MIS 8.5, at least (i.e.: *Equus hydruntinus*)^26^. The 403.0 ± 5.8 ka age obtained for the youngest population of crystals from sample OM-1 confirms continuous lacustrine deposition in the confined basin of Osteria Moricone 600 through 285 ka (i.e., the time span encompassing MIS 15 and MIS 8.5). The peculiar structural conditions which produced the isolated lacustrine basin of Osteria Moricone^26^ are illustrated in the map and cross-section of Suppl. Fig. #2-4b. These peculiar structural conditions allowed sedimentation within a small, isolated lacustrine basin overhanging the alluvial plain, and thus the elevation of the lacustrine deposits at Osteria Moricone should not be considered representative of the corresponding fluvial terraces in the Tiber Valley. We have re-assessed the geometry of the MIS 15 terrace based on the field observations in the main lacustrine basin at Ponte Sfondato to reconcile its elevation with that established at ca. 90 m a.s.l. At an elevation of ca. 85 m a.s.l. is a reworked volcaniclastic deposit dated at ≥541 ± 11 ka^26^ (Suppl. Fig. #2-4b), which should be considered a close approximation for the top elevation of the MIS 15 sedimentary succession. The reworked volcaniclastic deposit, based on its minimum age and litho-petrographic features, has been interpreted as a syn-eruptive, or shortly post-eruptive lahar deposit related to the Tufo Giallo della Via Tiberina pyroclastic-flow (546 ± 3 ka^11^). This eruption occurred during the early stages of the regressive phase following MIS 15 highstand. The pyroclastic-flow was likely deposited on a shallow erosional surface on top of the lacustrine succession of the Cretone basin, and may have eroded the volcanic succession of the previous eruption cycle (TGCP Eruption Cycle, 589 ± 4 ka^11^) that occurs on the MIS 15 terrace elsewhere (e.g. Ponte Sfondato). Evidence of strong post-sedimentary displacement of the MIS 15 terrace also occurs at Cretone (Suppl. Fig. #2-4b), where the MIS 15 deposits occur at ca. 70 m a.s.l., in the same sector where the Fall A and TGPP markers provide geometric constraints to the MIS 13 and MIS 11 terraces (Suppl. Fig. #2-4d). Further westward, close to the Tiber River Valley (i.e., Montelibretti Station, Suppl. Fig. #2-4g), the occurrence of the FAD-1 (589 ± 5 ka^11^) fallout deposit, which comformably overlies the alluvial plain as observed at Passo Corese, indicates an even lower elevation for the fluvial terrace of MIS 15 at ca. 30 m a.s.l.

The position and geometry of the buried faults responsible for the dislocation of the MIS 15 terrace are tentatively reconstructed in Suppl. Fig. #2-4, based on geomorphologic evidence and structural considerations related to the location of the Cretone Basin along the southern continuation of the N-S, strike-slip Sabina Fault zone^27,28^ (see Fig. 1). These fault segments, along with a main NE-SW transfer fault, should be considered as responsible for the origin of tectonic depressions which diverted the Tiber Valley from its NW-SE strike at the termination of the so-called Tiber Graben^3,29^ (Fig. 1).

### Monte Maggiore

Biostratigraphic data from the sedimentary succession cropping out at a quarry face located close to the Fara Sabina - Montelibretti railway station^30^, in the locality of Monte Maggiore (see Fig. 1b), provide further constraints to the MIS 5 terrace on the eastern bank of the Tiber Valley.

### Capena

**Figure 3:**
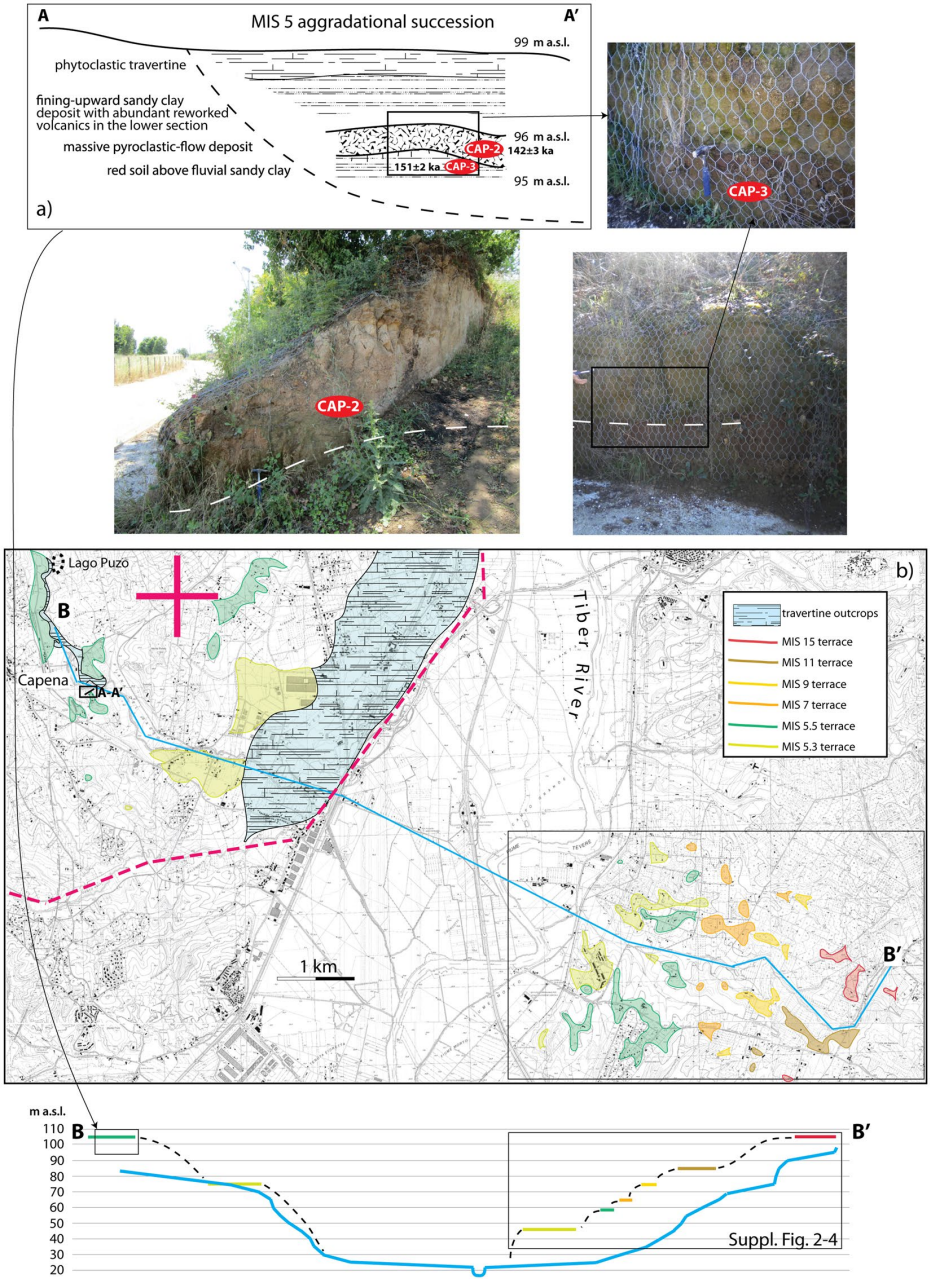
(**a**) A-A’: Stratigraphic scheme of the fluvial-lacustrine succession cropping out in Capena, showing the sampled volcanic (CAP-2) and sedimentary (CAP-3) deposits. (**b**) Morpho-structural map showing the suites of fluvial terraces on the two sides of the Tiber Valley. B-B’: transversal profile (blue line) showing the markedly different gradient affecting the western and eastern bank. Dashed red lines are the inferred faults bordering the uplifted sector (red cross).1:10.000 topographic base by Regione Lazio (http://dati.lazio.it/catalog/it/dataset/carta-tecnica-regionale-1991), available under Creative Commons Attribution License (https://creativecommons.org/licens/by/4.0/).

**Figure 4:**
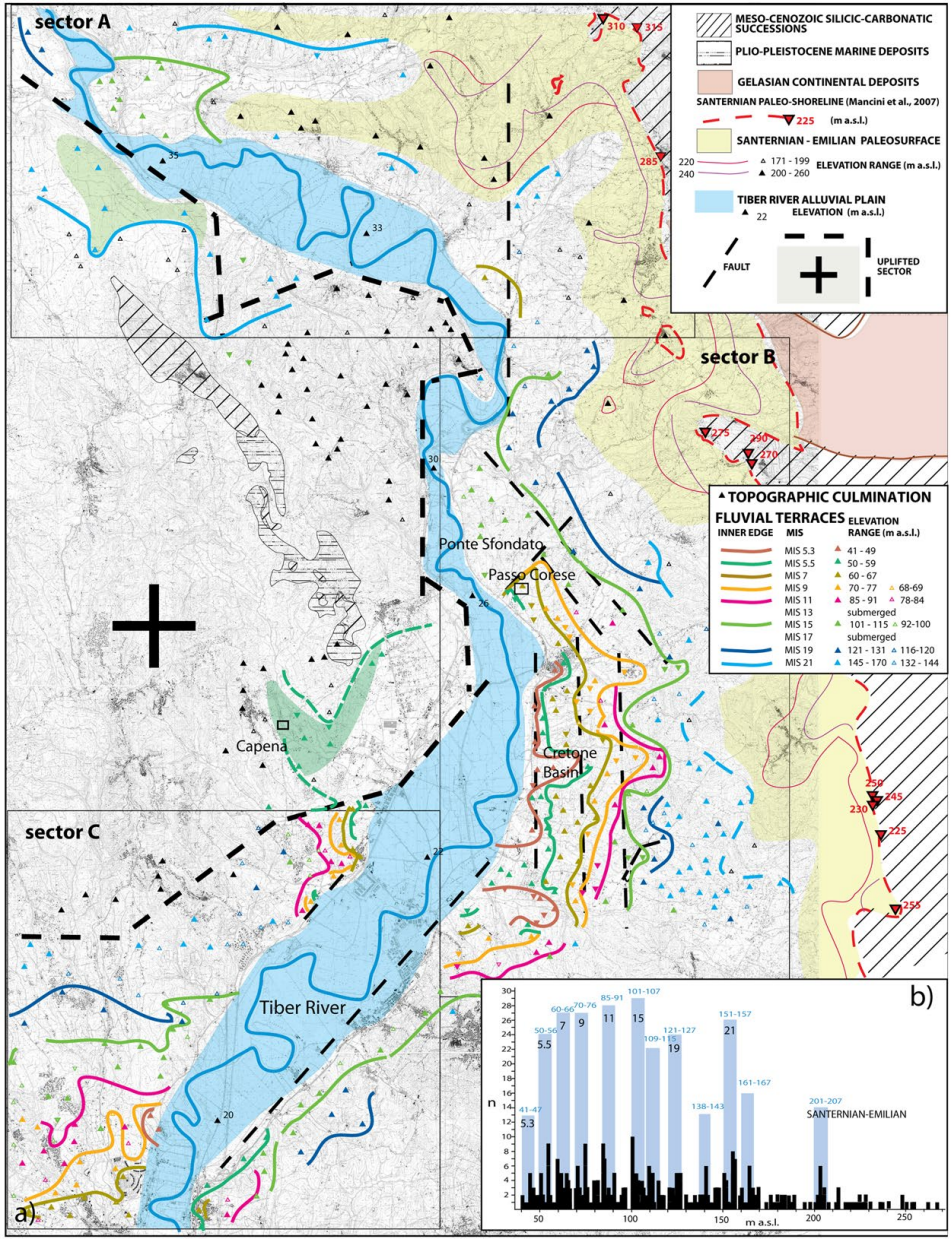
(**a**) Geomorphologic map of the investigated sector of the Tiber Valley. 1:10.000 topographic base by Regione Lazio (http://dati.lazio.it/catalog/it/dataset/carta-tecnica-regionale-1991), available under Creative Commons Attribution License (https://creativecommons.org/licens/by/4.0/). A series of terraced surfaces is reconstructed from a dataset of topographic culminations (colored triangles with upward vertex) in the area adjacent to the Santernian paleo-shoreline indicators^3^ (red triangles with downward vertex) following criteria established in^1,34^. The colored lines are the inner margin of terrace. The yellow shaded area represents the oldest paleo-surface corresponding to the top of the continental deposits of late Santernian-Emilian age. See text for further explanation. (**b**) Histograms reporting distribution and peaks of concentration for the topographic culminations for the total investigated area. A series of classes of elevation (vertical blue boxes), identifying the same number of paleo-surfaces are recognized and correlated with the sea-level highstands of MISs, following criteria described in the text.

An isolated, fragmented paleo-surface at around 105 m a.s.l. is located on the western bank of the Tiber Valley, near the town of Capena (Fig. 3), and is tentatively correlated with the MIS 15 terrace reconstructed in the eastern sector. A new field survey allowed us to verify that a fluvial-lacustrine succession, represented by whitish sandy muds, passing upward to travertine (Fig. 3b) underlies the paleo-surface culminating at 105 m a.s.l. However, the ^40^Ar/^39^Ar age of a pyroclastic-flow deposit at the base of the fluvial-lacustrine succession (sample CAP-2, Fig. 3b) yielded an unexpected young age of 141.8 ± 3 ka, ruling out the previous correlation with MIS 15. Moreover, a homogeneous population of crystals picked out from a sandy clay deposit underlying the pyroclastic-flow deposit (sample CAP-3, Fig. 3b) yielded an age of 151.0 ± 2.4 ka. These ages support correlation with two major eruptions from the nearby Vico Volcano; i.e., the C Ignimbrite (also known as Tufo Rosso a Scorie Nere from Vico or Sutri Formation^31^), dated at 151 ± 3–154 ± 1.5 ka, and the Vico D ignimbrite dated at 138 ± 2 ka^32^, and provide the straightforward correlation of the paleo-surface at 105 m a.s.l. with the MIS 5.5 terrace. This points out a markedly different elevation (+60 m) with respect to the MIS 5 terrace occurring at ca. 46 m a.s.l. on the western bank of the Tiber Valley (Fig. 4).

The MIS 5 deposits at Capena require a different interpretation with respect to the Cretone Basin, where very small, isolated outcrops of fluvial-lacustrine deposits dated within MIS 5 occur at higher elevation than the fluvial terrace bordering the Tiber Valley, and are interpreted as overhanging, tectonic basins. The San Martino stream valley, along which the terraced surface correlated with MIS 5.5 is exposed, widens towards the connection with the Tiber Valley, with no morpho-structural evidence of damming (Fig. 3b). Moreover, unlike the eastern tributary streams that are characterized by wide alluvial plains connected with an extremely low gradient to the main Tiber plain at 22–23 m a.s.l., the San Martino stream lacks an evident alluvial plain, cutting the western bank of the Tiber valley with a narrow and deep incision, characteristic of a steep gradient (see profile in Fig. 3b). Considering that the geologic substrate in the two sectors is the same (i.e., Plio-Pleistocene marine clay sediments), this geomorphological setting can be explained by a recent differential uplift affecting the western bank with respect to the eastern one, causing the marked difference in elevation of the MIS 5.5 and MIS 5.3 terraces in the two sectors. Notably, an extensive cover of thermogenic travertine^33^ exposed along the steeper western bank of the Tiber Valley suggests that rising hydrothermal fluids migrated through the preferential pathways represented by the faults and fractures bordering the uplifted sector, resulting in terraced steps declining towards the present alluvial plain.

### Hilltops elevations and related paleo-surfaces

A paleo-shoreline of Santernian age (i.e., 1.8–1.5 Ma), with decreasing altitude from ca. 315 m a.s.l. in the NW to ca. 225 m in the SE, is consistent with differential uplift due to the onset of Tuscan acid magmatism around 1.3 Ma^7^. This shoreline was reconstructed by^3^ along the western margin of Central Apennine, and in the Tiber Valley in the present work (Fig. 4). The highest paleo-surface ranging 201–220 m a.s.l. was interpreted as the oldest terrace in this area^1^, corresponding to an early coastal plain of late Santernian-Emilian age (1.6–1.3 Ma). The deposits forming this paleo-surface onlap onto Meso-Cenozoic carbonate structures of the Apennine, Gelasian continental deposits and Plio-Pleistocene marine sediments (Fig. 4). This paleosurface correlates to a tabular plateau at 250–270 m a.s.l. in the northernmost part of the Tiber Valley, formed by the continental deposits of the uppermost portion of the Chiani-Tevere Formation (Giove Formation), dating to 1.3 Ma according to^3^.

The oldest terrace is poorly preserved closer to the Tiber valley (Fig. 4), where a suite of younger terraced surfaces has been correlated by^1^ with the highstands of the sea level during MIS 21 through MIS 5, based on the principle of a staircase geometry and the available geochronologic constraints for the terraced lacustrine deposits of the Cretone Basin^26^. We have re-analyzed the hilltops dataset within the geographical area reported in Fig. 4, and slightly revised the statistics and mapping of the terrace inner edges in^1^, according to new field work and ^40^Ar/^39^Ar data, allowing us to refine the correlation of the paleo-surfaces with the MISs (Fig. 4b). For additional information on this refined correlation see Supplementary Material #1B.

### Comparison with DEM analysis

**Figure 5:**
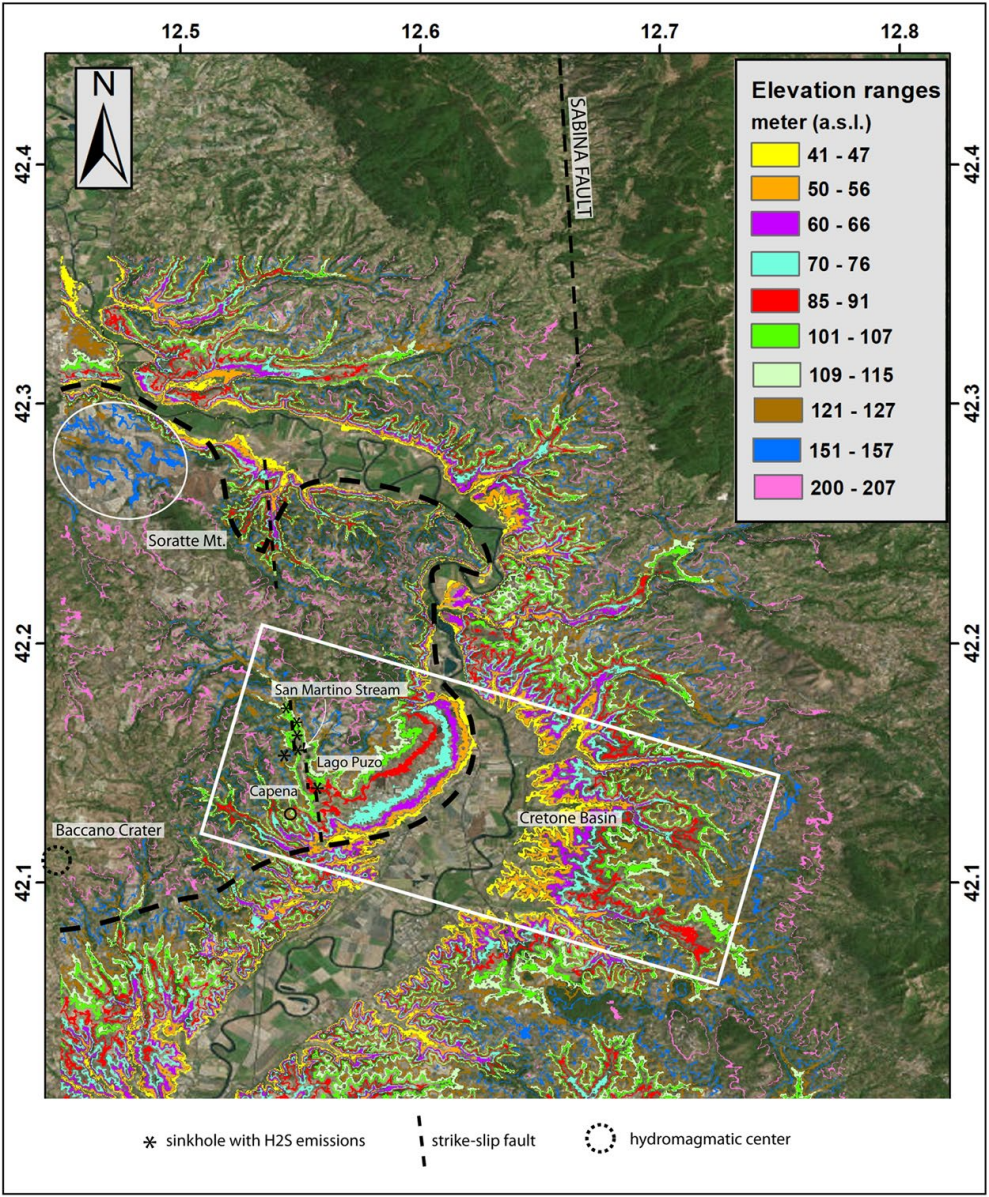
DEM of the investigated area highlighting eleven classes of elevations corresponding to those identified by the statistical analysis of hilltops elevation in Fig. 4b. Background image^44^ is a freeware by NASA shuttle mission, available under Creative Commons Attribution License (http://www2.jpl.nasa.gov/srtm): USGS (2004), Shuttle Radar Topography Mission, 1 Arc Second scene SRTM_u03_n008e004, Unfilled Unfinished 2.0, Global Land Cover Facility, University of Maryland, College Park, Maryland, February 2000.

Based on the statistical analysis of hilltop elevations (identified by the vertical blue boxes in Fig. 4b) eleven classes of elevations have been mapped. The DEM shows an average altitude accuracy of ~15 meters, nevertheless an overall coherent picture is highlighted by the image in Fig. 5, where the eleven classes of elevation depict a clear geomorphologic framework, despite each class ranging 6–7 m. However, only general considerations are possible, due to this limitation. Indeed, wideness of the colored sectors in Fig. 5 merely highlights the topographic gradients, allowing a comparison with the framework of paleo-surfaces reconstructed through the geomorphological study in Fig. 4.

A striking finding is the markedly different geomorphological settings on the two sides of the Tiber Valley. The eastern sector displays a wide and articulated drainage network and low gradients, consistent with the occurrence of a set of paleo-surfaces/fluvial terraces at progressively higher elevations (Fig. 4). In contrast, large part of the western bank of the Tiber Valley (bordered by the thick, dashed black line) is characterized by very steep gradients and lack of any terrace, consistent with the occurrence of recent uplift in this area. This is also evident in the rectangular area outlined in Fig. 5 comprising the Cretone Basin and the opposite valley bank, which are affected by differential uplift. The occurrence of the fluvial terraces along the eastern valley side is particularly well highlighted for the two lowest classes of elevations (yellow and orange), displaying a wide extension with respect to the opposite valley side, where the youngest terraces are missing and a peculiar geomorphologic setting is evidenced by the completely different trend of the colored bands.

The DEM image of Fig. 5 reveals a smoothed bulge, outlined by the concentric elevation bands around an elongated SW-NE half-dome connecting with a large plateau to the NW, and bordered by the N-S San Martino Stream Valley, where the MIS 5.5 terrace of 100–105 m occurs. A series of gas vents and sinkholes characterizes this stream valley^34^, which corresponds to a strike-slip tectonic lineament, part of the regional right-lateral, en-echelon Sabina Fault Zone^28^. Remarkably^33^, have highlighted the close relationships among the occurrence of thermogenic travertine deposits in the Tiber Valley, the major strike-slip tectonic lineaments, and phases of volcanic activity, particularly during MIS 5.

Another feature which is outlined by the DEM analysis is the very thin expression of the two highest classes of elevation at 151–157 m and 200–207 m, outlining the scarce preservation of the corresponding paleo-surfaces identified by the hilltops within these elevation ranges. The corresponding colored bands narrowly follow the contour lines, implying a lack of flat surfaces at the corresponding elevation range. One exception is in the area to the NW of Soratte Mt. encircled in Fig. 5, where the 151–157 m class of elevations has a widespread expression and is associated with topographic maximum at 165 m a.s.l. (Sector A, Fig. 4 and Suppl. Fig. #2-6b’). We interpret the terrace at 165–169 m to the NW of Soratte Mt (green shaded area in Fig. 4) as the uplifted equivalent of the MIS 15 terrace occurring at 100–105 m on the opposite valley bank.

## Discussion

### Assessment of the uplift curve

**Figure 6:**
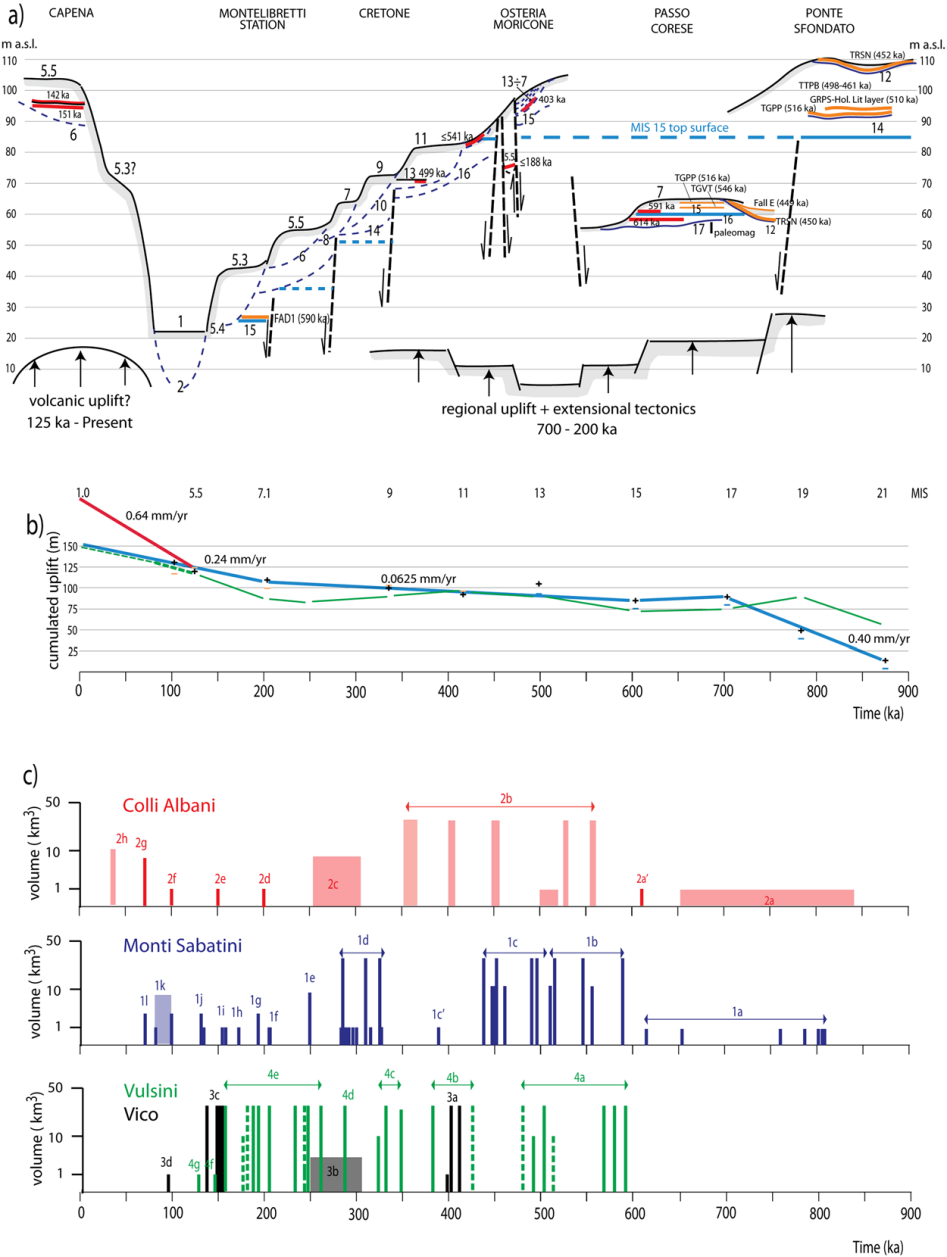
(**a**) Schematic, composite cross-section showing the morpho-stratigraphical and structural setting of the investigated area. Red and orange bars are volcanic layers dated or identified through their petrographic/componentry features, respectively, providing correlation between the aggradational successions and the highstands of the MISs (odd numbers associated with the fluvial terraces). The MIS 15 terrace, whose elevation has been pinpointed through the chronostratigraphic and geochronologic constraints achieved for the top surface of MIS 15 aggradational succession in Ponte Sfondato, Passo Corese, Cretone and Montelibretti Station, is shown (thick blue line). Deep blue lines are the erosive surfaces (dashed when not exposed) associated with the sea-level lowstands (even numbers). (**b**) Cumulated uplift curve in the time interval 900 ka through Present for the eastern side of the Tiber Valley (blue line), and in the last 125 ka for the western sector (red line). Black crosses are terrace elevations with respect to the MIS 1 terrace (assumed at 25 m a.s.l.), yellow and blue horizontal bars are the corrections for the maximum sea level with respect to Present after coral and speleothem record by^35^ for MIS 5 through MIS 13, and by assessment in^40^ for MIS 15 through 21, respectively. Comparison with the uplift curve for the coastal sector of Rome (thin green line) after^40^ is also provided. (**c**) Synoptic eruptive histories of the volcanic districts of the Roman Province based on ^40^Ar/^39^Ar and, subordinately, K/Ar age determinations from literature (see Supplementary Material for the detail of the eruptive phases and references). Boxes represent eruption cycles, bars represent single events. Dashed bars are known stratigraphic units lacking precise geochronologic constraints. Vertical height is proportional to estimated total erupted volumes in logarithmic scale (order of magnitude estimated from outcrop areas and crater/caldera size).

Based on new field data collected at Ponte Sfondato and the comparison with the stratigraphic setting of Cretone, we define the elevation of the MIS 15 terrace in the Tiber Valley at around 90 m a.s.l. in the sectors unaffected by differential fault displacement (Fig. 6a). Therefore, this value is used as a baseline for computations of the regional uplift that occurred in the last 600 ka, as represented by the blue curve of Fig. 6b. This curve is representative of the tectonic history of the area located to the east of the Tiber Valley, whereas a different curve (red color) denotes the sector to the west, based on the MIS 5 terrace data from Capena. Finally, the uplift curve assessed in^34^ for the coastal sector (green line) is also shown.

As discussed in^1^, a correction based on the different maximum sea-level estimations from the literature for each interglacial^35^ should be performed to assess the tectonic uplift. Moreover, a further correction should be introduced after evaluating the possible effect of glacial isostatic adjustment during each post-glacial period (e.g.^36^). This second estimate requires assessment of global ice volume during each glacial period, for which reliable indications are missing. Regarding absolute sea levels at past interglacials, their actual influence is uncertain, given the distance from the coast of the investigated sector and the continental feature of the terraced deposits. Also considering the tentative correlation with the MISs for the oldest terraces, we have considered the above-mentioned factors as negligible ones, in order to assess the general trend for the tectonic uplift, such as that depicted in Fig. 6b.

Based on field observations at Passo Corese, where the fluvial terrace of MIS 17 is below the younger MIS 15 terrace, we assume that this feature is the result of regional tectonics, rather than of local fault displacement. A similar terrace geometry and the occurrence of a thickened basal gravel succession correlated with MIS 17 in the coastal area of Ponte Galeria, is interpreted as a result of a wide collapse triggered by extensional tectonics^22^. Consequently, we revise previous correlation by^1^ who attributed the paleo-surface occurring at 125 m a.s.l. to the MIS 17 terrace (Fig. 6a) and we attribute it to MIS 19, while we correlate the overlying paleo-surface, characterized by a wide range of elevations spanning at 145–170 m, to the MIS 21 terrace. This wide range is likely the result of prolonged and widespread erosion affecting the oldest fluvial terrace, and may be due to the inclusion in the statistics of the sector to the NW of Soratte Mt., which is likely a younger, more recently uplifted terrace (Fig. 4). Therefore, we use the concentration peak at 151–156 m outlined in the eastern side of the Tiber Valley (Sector B in Suppl. Fig. #2-6b’’) as the elevation of the MIS 21 terrace.

We provide a small correction for the elevation of the MIS 19 and MIS 21 terraces because the thickness of the pyroclastic cover is much smaller than that mantling the MIS 15 paleo-surface, as inferred by the lack of volcanic deposits on the hilltops constraining the corresponding paleo-surfaces. Since the onset of the main explosive volcanic activity at ca. 600 ka, the graben-like morphology of the Tiber Valley likely resulted in the accumulation of primary and reworked pyroclastic deposits in the topographic lows rather than in overbank sectors. Therefore, we attribute the elevations of 120 and 155 m, respectively, to the MIS 19 and MIS 21 terraces.

### Implications on regional tectonics

When considered in the broader regional context, the terrace elevations correlated with MIS 21 through MIS 15 are consistent with the structural/stratigraphic framework of this sector of the Tyrrhenian Sea margin. The marked elevation gain between the terraces of MIS 21 and MIS 19 (ca. 35 m), mirrors that observed in the coastal area and is consistent with a pronounced pulse of regional uplift before 800 ka (Fig. 6b). This precedes the onset of volcanic activity in the area, which in turn was accompanied by intense episodes of extensional tectonics from 800 through 600 ka^37^ and references therein. In the coastal area the MIS 17 terrace overlies that of MIS 19^22^. Terrace geometry in the Tiber Valley (i.e., the lower elevation of the MIS 17 terrace) indicates that uplift persisted from MIS 19 through MIS 17, whereas tectonic subsidence occurred after MIS 19 in the coastal area (see Fig. 6b). Therefore, the extensional tectonics responsible for the lowering of the base level was delayed in the Tiber Valley, where intense faulting affected the MIS 17 and MIS 15 aggradational successions (Fig. 2b).

On the other hand, observation at Passo Corese indicates that, similar to the coastal setting, the MIS 15 terrace occurs slightly above that of MIS 17 also in the Tiber Valley north of Rome, suggesting that a subsiding phase characterized this time span along the whole Tyrrhenian Sea Margin of Latium (See Fig. 6b). The elevation of the MIS 13 terrace, constrained at 70 m in the Cretone Basin (Fig. 6a), suggests a minimum uplift of approximately 20 m between 600 and 530 ka, assuming similar maximum sea-levels during highstands of MIS 15 and MIS 13, as inferred from assessment for the sea-level of MIS 13 in^35^. However, the markedly low δ^18^O for MIS 13, coupled with the stratigraphic observations in Cretone and in Cava Rinaldi^38^ showing a lower paleo-sea level for this highstand with respect to MIS 11, suggests that in this case a relative sea-level correction is due (Fig. 6b).

Terrace elevations of MIS 11 through MIS 5 are assessed based on refined geomorphologic analysis in the Cretone Basin and from new biostratigraphic data from Monte Maggiore, and assuming a negligible thickness of volcanics, except for MIS 11 terrace, whose elevation is assessed at 83 m a.s.l., given the presence of a 2–3 m thick pyroclastic cover on the fluvial terrace (Suppl. Fig. #2-4b). Moreover, the occurrence of two marked peaks during MIS 5.5 and 5.3, separated by a ca. 25 ka time lapse (e.g.^39^), combined with the intervening uplift phase, supports correlation of the two paleo-surfaces at 55 and 46 m a.s.l. with the terraces of these two δ^18^O sub-stages. Consistently, a similar pair of terraced surfaces ranging 36 and 27 m a.s.l., has been identified and correlated with MIS 5.5 and 5.3 along the coast of Rome^40^.

### Comparison with the eruptive activity at the roman magmatic province

A steady, moderate uplift is highlighted in Fig. 6b between 600 and 200 ka, consistent with the sole isostatic component^1^, whereas a higher uplift rate occurred during the the last 200 ky, consistent with contribution of a new volcanic input at the level of the lower crust (i.e., astenosphere bulging^40^). The uplift rate during the last 125 ky is notably similar to that outlined on the coast assuming similar maximum sea-levels for MIS 5.5 and MIS 5.3 in the Mediterranean Sea (dashed green line in Fig. 6b; see^40^ for a discussion). A much higher uplift rate is required in the volcanic sector to the west of the Tiber valley in the same time span. Thus, the process of magma reservoir emplacement/changes in volume here affected the upper crust, and may be linked to the onset of the new phase of volcanic activity since ca. 130 ka.

Our new age determinations on three products of the most recent volcanic centers allow us to refine the eruptive history for the MSVD and to show that it lasted for a longer time span than previously thought, which shortens the time elapsed since the last documented eruption. Moreover, a climax for the last eruptive phase at 100–85 ka with the activity of at least six different centers is outlined (1k in Fig. 6c). MSVD activity is coeval with that of the Colli Albani Volcanic District (see^41^ for a discussion) as supported by the newly discovered last eruption at 70 ka, which is coincident with the first eruption cycle at the Albano Maar (1 l and 2 g in Fig. 6c).

The newly refined volcanic activity with the re-assessed uplift curves for the Tiber Valley north of Rome (blue and red lines in Fig. 6b) confirms previous assumptions of a marked uplift phase from 850 through 700 ka, coeval with the onset of the eruptive activity at Colli Albani and Monti Sabatini. Moderate subsidence characterizes the time span 700–600 ka, apparently coincident with a climax of extensional tectonics highlighted by intense faulting affecting the MIS 17 and MIS 16 aggradational successions in Passo Corese (Fig. 6a), and consistent with the start of the major phase of volcanic activity (Fig. 6c). A steady, moderate uplift characterizes the time span 700–200 ka, coincident with a prolonged period of explosive activity at four volcanic districts in Latium. In contrast, subsidence characterized the Tiber delta 350 through 200 ka (thin green line in Fig. 6b), possibly linked with progressive waning of the paroxysmal activity at Colli Albani and MSVD (Fig. 6c). A marked increase in the uplift rate occurs since 200 ka, which has been related by^40^ to the renewed energetic eruptive activity at MSVD and Colli Albani since 130 ka and 70 ka, respectively (Fig. 6c). Remarkably, an enhanced uplift rate (0.64 mm/yr) since 130 ka characterized the sector to the west of the Tiber Valley, corresponding to the eastern margin of the MSVD. However, it should be noted that the most outstanding evidence of deformation is localized on the western banks of the Tiber Valley, east of the San Martino Stream (Fig. 5), 20 km far from the area where the 130–70 ka activity occurred (Baccano and Martigano craters). Therefore, if the observed strong uplift is related with this volcanic phase, it affected the whole sector to the west of the Tiber Valley bordered by the dashed black line in Fig. 5.

We hypothesize that this tectonic event as driven by injection of magma into the upper crust, more locally concentrated with respect to those occurred 800 ka and 200 ka, likely reflecting the asthenosphere bulging linked with extensional tectonics and delamination processes affecting the Tyrrhenian Sea margin (e.g.^14^).

In general, there is a broad correlation between the uplift since 200 ka and the regional volcanic activity. However, it must be remarked that the eruptive volumes at the MSVD and Colli Albani increased during this time span, but the activity at Vico and Vulsini ceased by 100 ka and no sign of its possible rejuvenation has been reported so far (Fig. 6c), even though the uplift has affected the whole Latium coast in front of the volcanic region^42^. Future studies must focus on possible unrest in these volcanic districts and attempt to clarify the geodynamic factors driving the observed tectonic history on the Tyrrhenian Sea Margin of central Italy, including quantification of the isostatic, magmatic, and subduction-related contributions.

## Conclusions

As shown in Fig. 6c and in Suppl Mat. #3, we can identify three main volcanic periods. An ancient phase spans 850–650 ka, a later major explosive phase spans 600–450 ka, and a recent phase spans 320–70 ka.

Results of this study show:

(i) An increment in the waning eruptive activity since 320 ka occurred around 100 ka, when a number of different volcanic centers concentrated around the Baccano caldera produced high energetic, hydromagmatic eruptions^10^;
(ii) this recent activity persisted until 70 ka, when the last documented eruption occurred at the Martignano center, as ascertained in the present study;
(iii) the geomorphologic study performed for this work outlined an anomalous high elevation for the MIS 5 terrace along the bank of the Tiber Valley to the west of the most recently active volcanic area, which is the result of 50-m of uplift;
(iv) coupled geomorphologic and geochronologic constraints indicate that this uplift was concurrent with the most recent phase of volcanic activity spanning 100–70 ka, suggesting that it was induced by recharge of magma reservoirs;
(v) the dormancy since the last eruption (70 ky) exceeds the average recurrence interval (39 ky) of the last 300 ka, as well as the largest dormancy (50 ky) of this time span. It is of the same order as that separating the major explosive phase that occurred 590–450 ka at the Morlupo and Southern Sabatini centers and the subsequent period of large explosive eruptions of the Bracciano and Baccano centers after 320 ka.

While a large number of recent studies have ascertained the quiescent status of the nearby Colli Albani district, where the most recent eruption occurred 36 ka and where an ongoing inflation in the area hosting the most recent vents has been documented (ref.^43^ and references therein), no similar studies yet have been undertaken in the MSVD, preventing any assessment of current hazards. For this reason, a dedicated research project funded by INGV has been initiated, aimed at assessing possible active crustal deformation, including seismo-tectonic processes and vertical movements, in this volcanic region.

## Methods

### ^40^Ar/^39^Ar dating

Four volcanic deposits interbedded with the fluvial successions were sampled and dated by ^40^Ar/^39^Ar on single-crystals approach at the LSCE/Gif-sur-Yvette Laboratories, following procedures outlined in^44^ (see Suppl. Mat. #1 for full methods and data).

Three phreatomagmatic deposits erupted by the activity of Baccano, Martignano and Acquarello centers in the MSVD (BMU, MAR-3, ACQ) have been sampled and dated at the Wiscar Laboratory of the University of Wisconsin-Madison, following procedures described in^20^ (see Suppl. Mat. #1 for full methods and data).

### Paleomagnetic investigation

A ca. 1 m thick clay section exposed by quarry excavations at Passo Corese (Fig. 2) was sampled using 8-cm^3^ plastic cubes. In order to sample in fresh sediments, much effort was spent removing the surface. Twelve samples were taken with spacing ranging from 3 to 30 cm and were oriented with respect to vertical. The expected geocentric axial dipole field at the site latitude (about 42°N) has an inclination of ± 61° and of characteristic remanent magnetization (ChRM) inclinations are sufficient to determine the polarity. For full methods see Suppl. Material #1.

### Geomorphological analysis

In the present study we have re-assessed the elevations of the paleo-surfaces correlated by^1^ with the MISs in the southern area of investigation, by refining the geomorphologic study in several key sectors (i.e., Capena, Ponte Sfondato, Passo Corese, Cretone Basin, Fig. 1), integrating new field data and ^40^Ar/^39^Ar ages of 5 samples of volcanic deposits interbedded with the sedimentary successions. The employed geomorphological approach (see^1,34^ for the details) is based on the identification of topographic sectors characterized by flat hilltops whose elevations are comprised within a discrete interval of few meters, which identify corresponding paleo-surfaces. Statistical analysis of the hilltop elevations highlights peaks of concentration, representing the mean value for each paleo-surface.

### DEM analysis

The spatial analysis was performed into a GIS environment. Initially, an interferometric DEM with a ground resolution of 30 meters (1 arcsec) derived from the NASA SRTM mission (http://www2.jpl.nasa.gov/srtm)^45^ was cut on the basis of the study area using a polygon shapefile, then the various classes were identified one by one generating 11 different layers. To this aim, 11 queries were applied to the DEM, each time considering the maximum and minimum values of each class so to only isolate the desired altitude interval. Finally, the layers were merged into a single one and symbolized with 11 different colors, one for each class.

## Supplementary information

Supplementary Material #1

Supplementary Material 2

Supplementarty Material 3

## Data Availability

All data generated or analysed during this study are included in this published article.

## References

[1] Marra F, Florindo F, Petronio C (2017). Quaternary fluvial terraces of the Tiber Valley: geochronologic and geometric constraints on the back-arc magmatism-related uplift in central Italy. Journal Scientific Reports.

[2] Bridgland DR, Westaway R (2008). Climatically controlled river terrace staircases: a worldwide Quaternary phenomenon. Geomorphology.

[3] Mancini M, D’Anastasio E, Barbieri M, De Martini PM (2007). Geomorphological, paleontological and 87Sr/86Sr isotope analyses of early Pleistocene paleoshorelines to define the uplift of Central Apennines (Italy). Quaternary Research.

[4] Conticelli S, Peccerilllo A (1992). Petrology and geochemistry of potassic and ultrapotassic volcanism in central Italy: petrogenesis and inferences on the evolution of the mantle sources. Lithos.

[5] Serri G, Innocenti F, Manetti P (1993). Geochemical and Petrological evidence of the subduction of delaminated Adriatic continental lithosphere in the genesis of the Neogene-Quaternary magmatism of Central Italy. Tectonophysics.

[6] Peccerillo A., Frezzotti M. L. (2015). Magmatism, mantle evolution and geodynamics at the converging plate margins of Italy. Journal of the Geological Society.

[7] Barberi F (1994). Plio-Pleistocene geological evolution of the geothermal area of Tuscany and Latium. Mem. Descr. Carta Geol. d’ It..

[8] Karner DB, Marra F, Renne PR (2001). The history of the Monti Sabatini and Alban Hills volcanoes: groundwork for assessing volcanic-tectonic hazards for Rome. J. Volcanol. Geotherm. Res..

[9] Palladino, D. M., Simei, S., Sottili, G. & Trigila R. Integrated approach for the reconstruction of stratigraphy and geology of Quaternary volcanic terrains: An application to the Vulsini Volcanoes (central Italy), in: Groppelli, G. & Viereck-Goette, L., (Eds) *Stratigraphy and Geology of Volcanic Areas*. The Geological Society of America Special Paper, 464, 63–84, doi: 10.1130/2010.2464(04) (2010).

[10] Sottili G (2010). Geochronology of the most recent activity in the Sabatini Volcanic District, Roman Province, central Italy. Journal of Volcanology and Geothermal Research.

[11] Marra F (2014). Major explosive activity in the Sabatini Volcanic District (central Italy) over the 800-390 ka interval: geochronological - geochemical overview and tephrostratigraphic implications. Quaternary Science Reviews.

[12] Malinverno A, Ryan WBF (1986). Extension in the Tyrrhenian sea and shortening in the Apennines as results of arc migration driven by sinking of the lithosphere. Tectonics.

[13] Patacca, E., & Scandone, P. Post-Tortonian mountain building in the Apennines. The role of the passive sinking of a relic lithospheric slab, in *The Lithosphere in* Italy, edited by A. Boriani, M. Bonafede, G. B. Piccardo & G. B. Vai, Advances in Earth Science Research. It. Nat. Comm. Int. Lith. Progr., Mid-term Conf. (Rome, 5–6 May 1987). *Atti Conv*. *Lincei* **80**, 157–176 (1989).

[14] Jolivet L (1998). Midcrustal shear zones in postorogenic extension: Example from the northern Tyrrhenian Sea, *Jour*. Geoph. Res..

[15] Acocella V, Funiciello R (2006). Transverse systems along the extensional Tyrrhenian margin of central Italy and their influence on volcanism. Tectonics.

[16] Peccerillo, A. *Plio-Quaternary volcanism in Italy*. *Petrology*, *Geochemistry*, *Geodynamics*. Springer, Heidelberg (2005).

[17] Conticelli S, Avanzinelli R, Ammannati E, Casalini M (2015). The role of carbon from recycled sediments in the origin of ultrapotassic igneous rocks in the Central Mediterranean. Lithos.

[18] Luberti GM, Marra F, Florindo F (2017). A review of the stratigraphy of Rome (Italy) according to geochronologically and paleomagnetically constrained aggradational successions, glacio-eustatic forcing and volcano-tectonic processes. Quaternary International.

[19] Min K, Mundil R, Renne PR, Ludwig KR (2000). A test for systematic errors in ^40^Ar/^39^Ar geochronology through comparison with U/Pb analysis of a 1.1 Ga rhyolite. Geochimica et Cosmochimica Acta.

[20] Jicha BR, Singer BS, Sobol P (2016). Re-evaluation of the ages of ^40^Ar/^39^Ar sanidine standards and supereruptions in the western U.S. using a Noblesse multi-collector mass spectrometer. Chemical Geology.

[21] Channell JET, Hodell DA, Singer BS, Xuan C (2010). Reconciling astrochronological and ^40^Ar/^39^Ar ages for the Matuyama‐Brunhes boundary and late Matuyama Chron. Geochem. Geophys. Geosyst..

[22] Marra F, Florindo F, Karner DB (1998). Paleomagnetism and geochronology of early Middle Pleistocene depositional sequences near Rome: comparison with the deep sea δ^18^O climate record. Earth and Planetary Science Letters.

[23] Karner DB, Marra F (1998). Correlation of fluviodeltaic aggradational sections with glacial climate history: A revision of the Pleistocene stratigraphy of Rome. Geological Society of America Bulletin.

[24] Marra F, Florindo F, Boschi E (2008). The history of glacial terminations from the Tiber River (Rome): insights to glacial forcing mechanisms. Paleoceanography.

[25] Marra F (2016). Independent ^40^Ar/^39^Ar and 14C age constraints on the last five glacial terminations from the aggradational successions of the Tiber River, Rome (Italy). Earth Planet Sci Lett..

[26] Marra F (2016). Chronostratigraphic constraints on Middle Pleistocene faunal assemblages and Acheulian industries from the Cretone lacustrine basin, central Italy. Journal of Quaternary Science.

[27] Alfonsi L (1991). Structural and geochemical features of the Sabina strike-slip fault (Central Apennines). Boll. Soc. Geol. It..

[28] Faccenna C, Funiciello R, Mattei M, Late Pleistocene N-S (1994). shear zones along the Latium Tyrrhenian margin: structural characters and volcanological implications. Bollettino di Geofisica Teorica Applicata.

[29] Mancini M, Girotti O, Cavinato GP (2004). Il Pliocene e il Quaternario della Media Valle del Tevere. Geologica Romana.

[30] Angelelli, F. Descrizione e studio di resti di mammiferi del Plaistocene medio di Fara Sabina (Rieti-Lazio) conservati nelle collezioni del Servizio Geologico d’Italia. *Boll*. *Soc*. *geol*. *It*. **104**, 3–34 (1983–84).

[31] Perini G, Francalanci L, Davidson JP, Conticelli S (2004). Evolution and genesis of magmas from Vico volcano, Central Italy: multiple differentiation pathways and variable parental magmas. Journal of Petrology.

[32] Laurenzi MA, Villa IM (1987). ^40^Ar/^39^Ar chronostratigtaphy of Vico ignimbtites. Periodico di Mineralogia.

[33] Giustini F, Brilli M, Mancini M (2018). Geochemical study of travertinesaling middle-lower Tiber valley (central Italy): genesis, palaeo-environmental and tectonic implications. Int. J Earth Sci.

[34] Nisio S (2008). I sinkholes nel Lazio. Mem. Descr. Carta Geol. d’It..

[35] Rohling EJ (2009). Antarctic temperature and global sea level closely coupled over the past five glacial cycles. Nature Geoscience.

[36] Stocchi P (2018). MIS 5e relative sea-level changes in the Mediterranean Sea: Contribution of isostatic disequilibrium. Quaternary Science Reviews.

[37] Marra F, Florindo F (2014). The subsurface geology of Rome: sedimentary processes, sea-level changes and astronomical forcing. Earth-Science Reviews.

[38] Marra, F., Jicha, B. & Florindo, F. ^40^Ar/^39^Ar dating of Glacial Termination VI: constraints to the duration of Marine Isotopic Stage 13. *Scientific Reports* **7**, 8908, 10.1038/s41598-017-08614-6 10.1038/s41598-017-08614-6 PMC5566960 28827717

[39] Lisiecki LE, Raymo ME (2005). A Pliocene-Pleistocene stack of 57 globally distributed benthic δ^18^O records. Paleoceanography.

[40] Marra F, Florindo F, Anzidei M, Sepe V (2016). Paleo-surfaces of glacio-eustatically forced aggradational successions in the coastal area of Rome: assessing interplay between tectonics and sea-level during the last ten interglacials. Quaternary Science Reviews.

[41] Marra F (2004). Recurrence of volcanic activity along the Roman Comagmatic Province (Tyrrhenian margin of Italy) and its tectonic significance. Tectonics.

[42] Marra, F. *et al*. The archaeological ensemble from Campoverde (Agro Pontino, central Italy): new constraints on the Last Interglacial sea level markers, *Scientific Reports*, 10.1038/s41598-018-36111-x. (2018). 10.1038/s41598-018-36111-x PMC6292904 30546120

[43] Trasatti E., Marra F., Polcari M., Etiope G., Ciotoli G., Darrah T. H., Tedesco D., Stramondo S., Florindo F., Ventura G. (2018). Coeval Uplift and Subsidence Reveal Magma Recharging Near Rome (Italy). Geochemistry, Geophysics, Geosystems.

[44] Nomade S, Gauthier A, Guillou H, Pastre JF (2010). ^40^Ar/^39^Ar temporal framework for the Alleret maar lacustrine sequence (French Massif Central): Volcanological and Paleoclimatic implications. Quaternary Geochronology.

[45] Farr, T. G. *et al*. The Shuttle Radar Topography Mission, Proceedings of a Conference held 26-29 October 1999, Toulouse, France. European Space Agency, 2000. ESA-SP vol. 450, ISBN: 9290926414, p. 361 (2000).

