# Supplementary material for: Volcano-tectonic deformation in the Monti Sabatini Volcanic District at the gates of Rome (central Italy): evidence from new geochronologic constraints on the Tiber River MIS 5 terraces
Source: Sci Rep. 2019 Aug 8;9:11496. doi: 10.1038/s41598-019-47585-8 (PMC6687886; doi:10.1038/s41598-019-47585-8)
Supplement: Supplementary file 1 — Supplementary Material #1 [file 41598_2019_47585_MOESM1_ESM.pdf]

Volcano-tectonic deformation in the Monti Sabatini Volcanic District at the gates of Rome (central Italy): evidence from new geochronologic constraints on the Tiber River MIS 5 terraces

Marra, F.[1*], Florindo, F.[1], Jicha, B.[2], Nomade, S.[3], Palladino, D.M.[4], Pereira, A.[5,6], Sottili, G.[4], Tolomei, C.[1]

1) Istituto Nazionale di Geofisica e Vulcanologia, Via di Vigna Murata 605, 00143 Rome, Italy
2) Department of Geoscience, University of Wisconsin-Madison, USA
3) Laboratoire des Sciences du Climat et de l'Environnement, LSCE/IPSL, CEA-CNRS-UVSQ, Université Paris-Saclay, F-91191 Gif-sur-Yvette, France
4) Dipartimento di Scienze della Terra, "Sapienza" Università di Roma, Piazzale Aldo Moro 5, 00185 Roma, Italy
5) UMR 7194 HNHP MNHN-CNRS-UPVD, Départ. Homme et Environ. du MNHN,1 rue René Panhard, 75013
6) Ecole française de Rome, Piazza Farnese, IT-00186, Roma, Italy

*Corresponding author: fabrizio.marra@ingv.it

Supplementary Material #1A - Methods and datasets

Paleomagnetic analysis

First, the low field, volume specific, magnetic susceptibility (k) was measured for all samples on a Kappabridge KLY-4s Magnetic Susceptibility Meter (AGICO Inc. Brno, Czech Republic). The natural remanent magnetization (NRM) was then analyzed within the magnetically shielded paleomagnetic laboratory at the Istituto Nazionale di Geofisica e Vulcanologia, Rome, using a pass-through 2-G Enterprises magnetometer equipped with in-line alternating field (AF) demagnetization capability. The NRM was stepwise AF demagnetized at successive peak fields of 5, 10, 15, 20, 30, 40, 50, 60, 70, 80 and 100 mT and demagnetization data were examined using orthogonal vector component diagrams. ChRM components were determined from principal component analysis and the precision related to each best-fit line was estimated by the maximum angular deviation (MAD). In order to determine the characteristic Curie or Néel temperatures of magnetic minerals we continuously monitored the temperature dependence of k using a CS-3 furnace equipped Kappabridge KLY-3 system [1] on a sample characterised by the highest k and NRM (located at 22 cm).

$^{40}Ar/^{39}Ar$ dating

LSCE

After sample crushing and sieving, potassium feldspars crystals (i.e., Sanidine) ranging from 400 μm to up to 1 mm in size were individually handpicked under a binocular and leached for 5 min in 7% HF acid. About thirty minerals were finally chosen after leaching and separately loaded in a single aluminium disk for each sample. Samples were irradiated for 90 min (IRR 109) in the β1 tube of the OSIRIS reactor (French Atomic Energy Commission, Saclay, France).

After irradiation, the feldspars were transferred into a copper sample holder and loaded individually into a vacuum Cleartran© window. Crystals for each sample were individually fused using a Synrad CO_2 at 10-15 % nominal power (c.a 25 W). The Ar isotopes were measured using a VG5400 mass spectrometer equipped with a single ion counter (Balzers© SEV 217 SEN). Neutron fluence (*J*) was monitored by co-irradiation of Alder Creek sanidines standard (ACs-2) [2]. *J* value for each sample was determined from analyses of two ACs-2 single crystal measurements. *J* values were calculated using ACs-2 at 1.193 Ma [2], the total decay constant of [3] and the $^{40}Ar/^{36}Ar$ atmospheric ratio of 298.56 [4]. For an easier intercomparison, ages obtained are in Table 1A, also recalculated using the decay constant of [5] and the recommended age by Niespolo et al., 2017 for ACs-2 (1.185 Ma). Procedural blanks were measured every two or three unknowns depending of the bean size. For a typical 10-min static blank, the backgrounds were generally about 2.0–2.2 × 10^{-17} and 5.0–6.0 × 10^{-19} mol for ^{40}Ar and ^{36}Ar, respectively. The precision and accuracy of the mass discrimination correction was monitored by weekly measurements of various beam sizes of atmospheric air (see [6] for full experimental description).
For all the samples, $^{40}Ar/^{39}Ar$ results are presented as probability diagrams, Neutron fluence (J) values could be found for each sample in the supplementary material (Tables S1 to S5). A population of crystals was considered relevant if the weighted mean age of the considered population presents the following statistical characteristics: mean square weighted deviation (MSWD) < 2.0 and a probability fit (P) > 0.1. Weighted averages are calculated using Isoplot 3.0 [7] and given at 2σ analytical uncertainties.

40Ar/39Ar Wiscar Laboratory

Sanidine phenocrysts were isolated from sample MG4 using standard magnetic and density separation techniques, and were co-irradiated with the 1.1864 Ma Alder Creek sanidine standard [8, 9] at the Oregon State University TRIGA reactor in the Cadmium-Lined In-Core Irradiation Tube. Single crystal fusion analyses were performed at the WiscAr laboratory at the University of Wisconsin-Madison using a 60W CO_2 laser and a Noblesse multi-collector mass spectrometer following [8]. Results are reported in Table 1B, full dataset in Table S6.

Supplementary Material #1B - Sample descriptions

CAP-2 sample

The sampled unit is a massive pyroclastic-flow deposit, dark brown in color, ca. 1 m thick. It is constituted by mm to cm-sized dark scoriae and lapilli within an ash matrix, including abundant loose pyroxene and sanidine crystals. The probability diagram provided for CAP-2 is unimodal. The weighted mean age calculated for the juvenile population is 141.8±3.0 ka (Figure S1-1) with a MSWD of 0.95. The probability calculated is 0.49. The $^{40}Ar/^{36}Ar$ initial ratio is imprecise (274 ± 47 (2σ)), but equivalent within uncertainties to the atmospheric ratio of 298.56 [4]. No argon excess is thus highlighted.

CAP-3 sample

Loose sanidine crystals have been picked from a sedimentary, sandy clay deposit reddish in color, underlying the pyroclastic-flow deposit from which sample CAP-2 was collected.
The probability diagram is unimodal like CAP-2 and is characterized by an homogeneous population of juvenile crystals allowing us to calculate a weighted mean age of 151.0 ± 2.4 ka, MSWD = 0.81 and P =0.62. The related $^{40}Ar/^{36}Ar$ initial ratio of 299.0 ± 2.0 (2σ) is equivalent within uncertainties to the atmospheric ratio of 298.56 [4].

OM-1 sample

Sanidine crystals have been extracted from a volcaniclastic deposit, constituted by mm-sized dark scorie, and loose pyroxenes, sanidine and analcimized leucite crystals, adhering to a bifacial stone tool recovered within the lacustrine deposits cropping out in Osteria Moricone.
The probability diagram obtained is multimodal and evidences two main crystal populations: the main one, centered around 455 ka, and the youngest population, composed by four crystals, is associated to a weighted mean age of 403.0 ± 5.8 ka, MSWD = 1.06 and P =0.36. The corresponding $^{40}Ar/^{36}Ar$ initial ratio of 296.0 ± 10.0 (2σ) for the youngest population is equivalent within uncertainties to the atmospheric ratio of 298.56 [4].

PC-6 sample

This sample was collected from a bedded white and light grey pumice fall deposit, ca. 1 m thick, cropping out at the Passo Corese section (pu1 in Figure 2a). The probability diagram obtained for PC-6 is simple, with one main population excluding 2 crystals. The main mode, including 8 out of 10 sanidine crystals measured allows to calculate a straightforward age of

591.3 ± 2.6 ka, MSWD = 1.17 and P =0.31. The related $^{40}Ar/^{36}Ar$ initial ratio of 300.0 ± 2.0 (2σ) is equivalent within uncertainties to the atmospheric ratio of 298.56 [4].

PC-5 sample

The sample unit is a dark grey, massive pyroclastic-flow deposit, 10 to 30 cm thick. It is constituted by mm-sized dark scoriae and loose crystals within a faintly laminated ash matrix. The probability diagram obtained for PC-5 is multimodal, as at least three populations are found (see Figure S1-1). The youngest and probably juvenile population, including 5 out of 11 measured sanidine crystals, allows to calculate a weighted mean age of 614.3 ± 3.4 ka, MSWD = 1.13 and P = 0.34. The related $^{40}Ar/^{36}Ar$ initial ratio of 299.0 ± 10.0 (2σ) is equivalent within uncertainties to the atmospheric ratio of 298.56 [4].

BMU sample

The sampled unit is a hydromagmatic deposit constituted by a light yellow, massive, lithified ash matrix, including sparse, cm-sized sedimentary and volcanic lithics and abundant, sub-mm sized loose pyroxene crystals. Scattered age results evidence the occurrence of abundant xenocrystals, consistent with the hydromagmatic features of this eruptive unit (Table S6). However, a statistically significant youngest population of six crystals constrains eruption age at 99.3 ± 2.7 ka.

MAR-3 sample

The sampled unit is a pyroclastic surge deposit constituted by a light grey ash matrix with sparse accretionary lapilli, cm-sized volcanic and carbonate clasts, and abundant loose leucite, feldspar, clinopyroxene and biotite crystals. The prevalent magmatic features of this eruption unit are inferred by the single population of crystals (Table S6), evidencing a fully juvenile sampled fraction of 70.0 ± 3.3 ka.

ACQ sample

The sampled unit is a hydromagmatic deposit constituted by abundant sedimentary and volcanic lithics, subordinated juvenile (?) scoriae, and loose crystals, within a strongly lithified, dark yellow ash matrix. Consistently, sample ACQ contains widespread contamination by older crystals and a juvenile youngest crystal population of 82.5±4.4 ka (Table S6).

REFERENCES

1) Hrouda, F. A technique for the measurements of thermal changes of magnetic susceptibility of weakly magnetic rocks by the CS-2 Apparatus and KLY-2 Kappabridge. *Geophys. J. Int.* **118**, 604–612, doi:10.1111/j.1365-246X.1994.tb03987.x (1994).

2) Nomade, S. *et al.* Alder Creek sanidine (ACs-2), A Quaternary $^{40}Ar/^{39}Ar$ dating standard tied to the Cobb Mountain geomagnetic event. *Chemical Geology* **218**, 315-338 (2005).

3) Steiger, R. H. & Jäger, E. Subcommission on geochronology: convention on the use of decay constants in geo- and cosmo- chronology. *Earth and Planetary Science Letters* **36** (3), 359-362 (1977).

4) Lee, J. Y. *et al*. A redetermination of the isotopic abundances of atmospheric Ar. *Geochimica et Cosmochimica Acta* **70**, 4507-4512, doi: 10.1016/j.gca.2006.06.1563 (2006).

5) Min, K., Mundil, R., Renne, P.R. & Ludwig K. R. A test for systematic errors in 40Ar/39Ar geochronology through comparison with U/Pb analysis of a 1.1 Ga rhyolite. *Geochimica et Cosmochimica Acta* **64** (1), 73-98 (2000).

6) Nomade, S., Gauthier, A., Guillou, H. & Pastre, J. F. $^{40}Ar/^{39}Ar$ temporal framework for the Alleret maar lacustrine sequence (French Massif Central): Volcanological and Paleoclimatic implications, *Quaternary Geochronology* **5,** 20-27 (2010).

7) Ludwig, K. R. Isoplot 3.0a Geochronological Toolkit for Microsoft Excel. In Special Publication No. 4. Berkeley Geochronology Center: Berkeley, CA (2001).

8) Jicha, B. R., Singer, B. S. & Sobol, P. Re-evaluation of the ages of 40Ar/39Ar sanidine standards and supereruptions in the western U.S. using a Noblesse multi-collector mass spectrometer, *Chemical Geology* **431**, 54–66 (2016).

9) Rivera, T. A., Storey, M., Schmitz, M. D. & Crowley, J. L. Age intercalibration of $^{40}Ar/^{39}Ar$ sanidine and chemically distinct U/Pb zircon populations from the Alder Creek Rhyolite Quaternary geochronology standard. *Chemical Geology* **345**, 87-98 (2013).

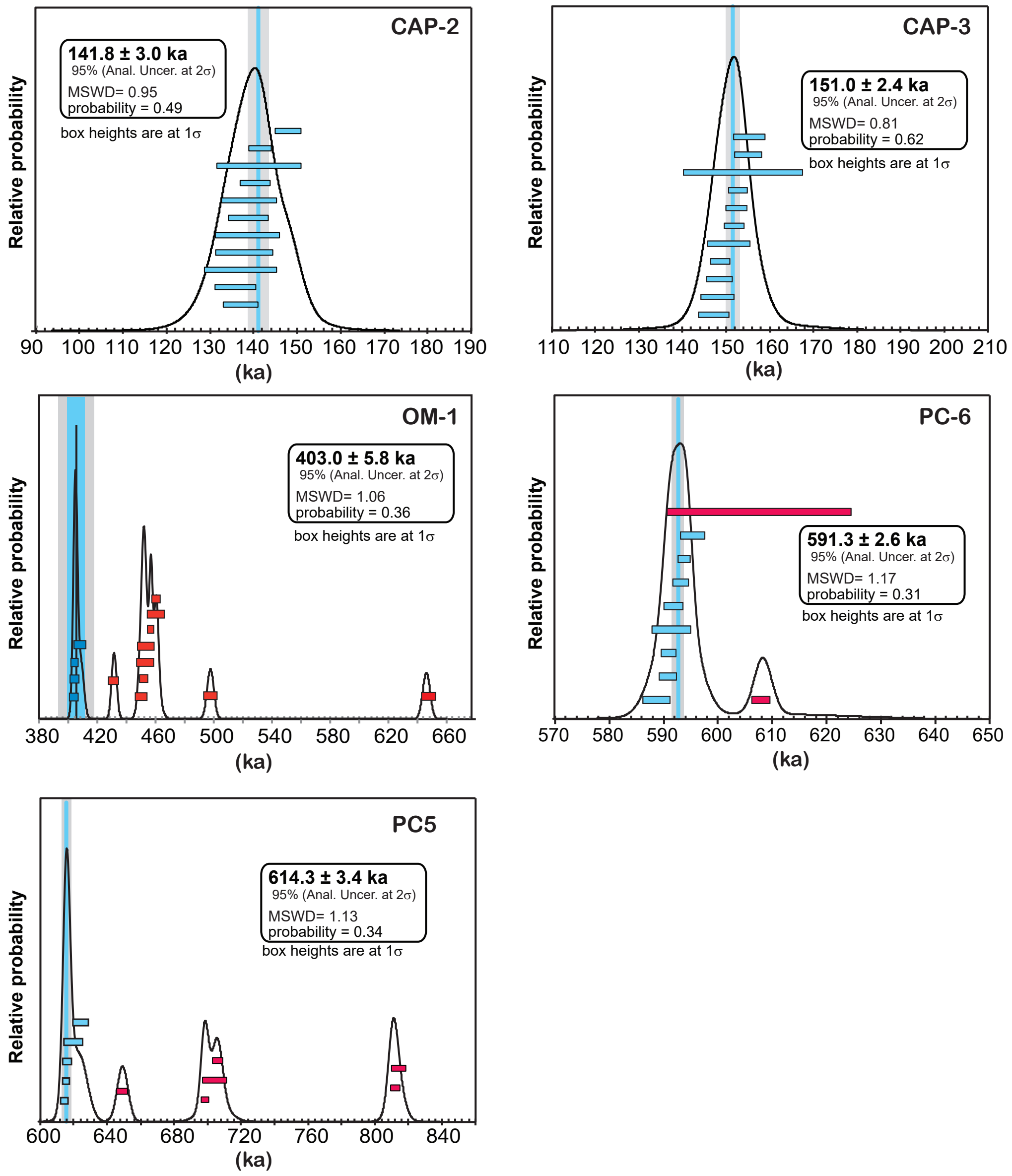

*Ages related to the K total decay constant of Min et al., 2000 and the flux-standard ACs at 1.1864 Ma **(in Figure caption)***

Figure S1

Sample ID:	**CAP 2**	Lab# N1562-01/N1562-11	J = 0.00039170 ± 0.00000196	
Sanidine		Irradiation # 109	reactor	OSIRIS
Flux standard	ACs-2	1.193 Ma	Single crystal total fusion	

Ages reported in the following supplementary dataset are calculated according to the K total decay constant of Steiger and Jäger, (1977) and the flux standard Acs-2 dated to 1.193 Ma

N	^{40}Ar	^{36}Ar	±% σ_{36}	^{37}Ar	±%σ_{37}	^{38}Ar	±%σ_{38}	^{39}Ar	±%σ_{39}	^{40}Ar	±%σ_{40}	D(1)	±%σ_D	%^{40}Ar*	Age	±σ	K/Ca	± 1σ
	(moles)	V		V		V		V		V					(ka)	(ka)		
**N1562-01**	**7,692E-16**	**4,20E-07**	**5,8**	**6,74E-05**	**241,9**	**3,65E-05**	**0,5**	**2,23E-03**	**0,2**	**5,61E-04**	**0,2**	**1,009**	**0,07**	**79,24**	**140,7**	**± 6,3**	**14,3**	**± 34,5**
**N1562-02**	**6,706E-16**	**2,69E-07**	**7,6**	**6,74E-05**	**241,9**	**3,43E-05**	**0,5**	**2,11E-03**	**0,2**	**4,89E-04**	**0,2**	**1,009**	**0,07**	**85,34**	**139,7**	**± 6,5**	**13,5**	**± 32,6**
**N1562-03**	**3,206E-16**	**4,96E-08**	**30,1**	**4,70E-05**	**235,9**	**1,82E-05**	**0,7**	**1,15E-03**	**0,2**	**2,34E-04**	**0,3**	**1,009**	**0,07**	**96,14**	**138,8**	**± 8,2**	**10,5**	**± 24,7**
**N1562-04**	**7,543E-16**	**3,41E-07**	**4,3**	**4,83E-05**	**256,8**	**3,91E-05**	**0,6**	**2,29E-03**	**0,2**	**5,51E-04**	**0,2**	**1,009**	**0,07**	**82,77**	**140,6**	**± 4,5**	**20,4**	**± 52,4**
**N1562-05**	**8,151E-16**	**3,33E-07**	**5,6**	**7,02E-05**	**168,1**	**3,68E-05**	**0,2**	**2,59E-03**	**0,2**	**5,95E-04**	**0,2**	**1,009**	**0,07**	**84,63**	**137,4**	**± 4,0**	**15,9**	**± 26,7**
**N1562-06**	**6,468E-16**	**3,09E-07**	**4,7**	**3,57E-05**	**483,9**	**3,46E-05**	**0,6**	**1,94E-03**	**0,1**	**4,72E-04**	**0,2**	**1,009**	**0,07**	**81,68**	**140,4**	**± 7,3**	**23,4**	**± 113,2**
**N1562-07**	**6,295E-16**	**1,78E-07**	**7,4**	**7,95E-05**	**308,4**	**3,53E-05**	**0,5**	**2,06E-03**	**0,2**	**4,60E-04**	**0,2**	**1,009**	**0,07**	**90,70**	**143,0**	**± 9,6**	**11,1**	**± 34,4**
**N1562-08**	**1,223E-15**	**7,37E-07**	**2,6**	**9,59E-05**	**206,0**	**5,69E-05**	**0,5**	**3,52E-03**	**0,1**	**8,93E-04**	**0,2**	**1,009**	**0,07**	**76,74**	**137,7**	**± 4,6**	**15,8**	**± 32,5**
**N1562-09**	**1,405E-15**	**7,80E-07**	**2,4**	**1,01E-04**	**114,8**	**6,34E-05**	**0,3**	**3,98E-03**	**0,1**	**1,03E-03**	**0,1**	**1,009**	**0,07**	**78,57**	**143,2**	**± 2,5**	**16,9**	**± 19,4**
**N1562-10**	**8,033E-16**	**1,05E-07**	**11,6**	**1,01E-04**	**114,8**	**4,66E-05**	**0,4**	**2,82E-03**	**0,1**	**5,86E-04**	**0,1**	**1,009**	**0,07**	**96,86**	**142,1**	**± 3,4**	**12,0**	**± 13,8**
**N1562-11**	**7,424E-16**	**6,53E-08**	**16,8**	**4,44E-05**	**194,6**	**4,57E-05**	**0,4**	**2,50E-03**	**0,2**	**5,42E-04**	**0,2**	**1,009**	**0,07**	**97,78**	**149,6**	**± 2,9**	**24,2**	**± 47,1**

Results	40Ar*/39Ark ± 1σ	Age ± 1σ (Ka)	MSWD	39Ar(k) (n)	K/Ca ± 1σ
Weighted mean	0,2018 ± 0,0018 ± 0,90%	142,6 ± 1,5 ± 1,03%	0,95	100,00 11	14,0 ± 8,0
		Full External Error ± 2,3	0,98	Statistical T ratio	
		Analytical Error ± 1,3	1,0000		

Results	40(a)/36(a) ± 1σ	40(r)/39(k) ± 1σ	Age ± 1σ (ka)	MSWD
Inverse Isochron	274,4966 ± 23,6412 ± 8,61%	0,2044 ± 0,0030 ± 1,45%	144,4 ± 2,2 ± 1,53%	0,96
Full External Error			± 2,8	
Analytical Error			± 2,1	

Statistics				
	Statistical F ratio	1,11	Convergence	0,0002319742
	Error Magnification	1,0000	Number of Iterations	3
	Number of Data Points	11	Calculated Line	Weighted York-2

Background corrections CAP 2

N	^{36}Ar	±σ36	^{37}Ar	±σ37	^{38}Ar	±σ38	^{39}Ar	±σ39	^{40}Ar	±σ40
	V	V	V	V	V	V	V	V	V	V
N1562-01	1,01E-07	1,49E-08	4,78E-08	9,84E-09	8,16E-08	1,88E-08	1,22E-07	1,21E-07	1,45E-05	6,53E-07
N1562-02	1,01E-07	1,49E-08	4,78E-08	9,84E-09	8,16E-08	1,88E-08	1,22E-07	1,21E-07	1,45E-05	6,53E-07
N1562-03	6,89E-08	6,65E-09	3,94E-08	5,71E-09	1,39E-08	1,38E-08	2,66E-06	1,34E-06	1,46E-05	4,08E-07
N1562-04	6,89E-08	6,65E-09	3,94E-08	5,71E-09	1,39E-08	1,38E-08	2,66E-06	1,34E-06	1,46E-05	4,08E-07
N1562-05	6,89E-08	6,65E-09	3,94E-08	5,71E-09	1,39E-08	1,38E-08	2,66E-06	1,34E-06	1,46E-05	4,08E-07
N1562-06	7,25E-08	7,25E-09	2,97E-08	1,25E-08	2,92E-08	2,89E-08	5,07E-07	2,13E-07	1,55E-05	2,79E-07
N1562-07	7,25E-08	7,25E-09	2,97E-08	1,25E-08	2,92E-08	2,89E-08	5,07E-07	2,13E-07	1,55E-05	2,79E-07
N1562-08	7,25E-08	7,25E-09	2,97E-08	1,25E-08	2,92E-08	2,89E-08	5,07E-07	2,13E-07	1,55E-05	2,79E-07
N1562-09	8,24E-08	9,31E-09	2,34E-08	5,15E-09	1,91E-08	1,89E-08	3,65E-07	1,38E-07	1,57E-05	2,82E-07
N1562-10	8,24E-08	9,31E-09	2,34E-08	5,15E-09	1,91E-08	1,89E-08	3,65E-07	1,38E-07	1,57E-05	2,82E-07
N1562-11	1,29E-07	6,84E-09	2,11E-08	4,59E-09	1,77E-08	1,65E-08	9,33E-07	5,13E-08	2,29E-05	4,34E-07

Table S1

Table S1

Sample ID:	**CAP 3**	Lab# N1563-01/N1563-11	J = 0.00038750 ± 0.00000194	
Sanidine		Irradiation #109	reactor	OSIRIS
Flux standard	ACS-2	1.193 Ma	Single crystal total fusion	

N	^{40}Ar	^{36}Ar	±% σ_{36}	^{37}Ar	±%σ_{37}	^{38}Ar	±%σ_{38}	^{39}Ar	±%σ_{39}	^{40}Ar	±%σ_{40}	D(1)	±%σ_D	%^{40}Ar*	Age	±σ	K/Ca	± 1σ
	(moles)	V		V		V		V		V					(ka)	(ka)		
**N1563-01**	**2,434E-15**	**4,04E-06**	**0,395**	**1,36E-04**	**77,632**	**4,61E-05**	**0,540**	**2,77E-03**	**0,139**	**1,78E-03**	**0,124**	**1,009**	**0,07**	**32,99**	**147,8**	**± 3,5**	**8,8**	**± 6,8**
**N1563-02**	**1,046E-15**	**8,60E-08**	**7,818**	**1,34E-04**	**71,128**	**5,04E-05**	**0,388**	**3,53E-03**	**0,106**	**7,64E-04**	**0,155**	**1,009**	**0,07**	**98,64**	**149,2**	**± 2,2**	**11,3**	**± 8,0**
**N1563-03**	**3,030E-15**	**3,73E-06**	**0,675**	**9,51E-05**	**126,309**	**8,56E-05**	**0,305**	**5,10E-03**	**0,130**	**2,21E-03**	**0,112**	**1,009**	**0,07**	**50,26**	**152,5**	**± 2,2**	**23,0**	**± 29,1**
**N1563-04**	**1,291E-15**	**1,71E-07**	**13,064**	**1,42E-04**	**65,683**	**6,53E-05**	**0,425**	**4,15E-03**	**0,130**	**9,42E-04**	**0,169**	**1,009**	**0,07**	**96,46**	**153,3**	**± 2,1**	**12,6**	**± 8,2**
**N1563-05**	**1,414E-15**	**6,41E-07**	**3,122**	**4,37E-05**	**311,902**	**5,48E-05**	**0,434**	**3,80E-03**	**0,148**	**1,03E-03**	**0,135**	**1,009**	**0,07**	**81,97**	**155,6**	**± 3,1**	**37,4**	**± 116,6**
**N1563-07**	**1,172E-15**	**3,19E-07**	**5,025**	**4,57E-05**	**362,235**	**5,68E-05**	**0,539**	**3,61E-03**	**0,148**	**8,55E-04**	**0,123**	**1,009**	**0,07**	**89,65**	**148,6**	**± 3,7**	**33,9**	**± 122,9**
**N1563-08**	**1,065E-15**	**3,60E-07**	**3,848**	**6,68E-05**	**280,185**	**4,86E-05**	**0,521**	**3,14E-03**	**0,139**	**7,78E-04**	**0,154**	**1,009**	**0,07**	**87,28**	**151,2**	**± 4,8**	**20,2**	**± 56,6**
**N1563-09**	**2,265E-14**	**5,21E-05**	**0,397**	**1,05E-04**	**131,070**	**8,28E-05**	**0,305**	**4,42E-03**	**0,122**	**1,65E-02**	**0,060**	**1,014**	**0,07**	**5,91**	**154,4**	**± 13,5**	**18,1**	**± 23,7**
**N1563-10**	**1,224E-15**	**2,18E-07**	**7,163**	**5,97E-05**	**178,192**	**6,66E-05**	**0,360**	**3,83E-03**	**0,139**	**8,93E-04**	**0,196**	**1,009**	**0,07**	**93,81**	**153,0**	**± 2,4**	**27,6**	**± 49,2**
**N1563-11**	**1,643E-15**	**1,75E-06**	**1,427**	**9,87E-05**	**123,039**	**5,20E-05**	**0,256**	**3,10E-03**	**0,130**	**1,20E-03**	**0,124**	**1,009**	**0,07**	**57,65**	**155,9**	**± 3,6**	**13,5**	**± 16,6**

Results	40Ar*/39Ark ± 1σ	Age ± 1σ (Ka)	MSWD	39Ar(k) (n)	K/Ca ± 1σ
Weighted mean	0,2171 ± 0,0012 ± 0,58%	151,8 ± 1,2 ± 0,76%	0,81	100,00 11	11,6 ± 4,1
		Full External Error ± 2,2	0,98	Statistical T ratio	
		Analytical Error ± 0,9	1,0000		

Results	40(a)/36(a) ± 1σ	40(r)/39(k) ± 1σ	Age ± 1σ (ka)	MSWD
Inverse Isochron	298,6897 ± 1,1745 ± 0,39%	0,2171 ± 0,0013 ± 0,60%	151,7 ± 1,2 ± 0,78%	0,92
Full External Error			± 2,2	
Analytical Error			± 0,9	

Statistics				
	Statistical F ratio	1,11	Convergence	0,0008388667
	Error Magnification	1,0000	Number of Iterations	2
	Number of Data Points	11	Calculated Line	Weighted York-2

Background corrections CAP 3

N	^{36}Ar	±σ36	^{37}Ar	±σ37	^{38}Ar	±σ38	^{39}Ar	±σ39	^{40}Ar	±σ40
	V	V	V	V	V	V	V	V	V	V
N1563-01	1,29E-07	6,84E-09	2,11E-08	4,59E-09	1,77E-08	1,65E-08	9,39E-07	5,16E-08	2,29E-05	4,34E-07
N1563-02	1,29E-07	6,84E-09	2,11E-08	4,59E-09	1,77E-08	1,65E-08	9,39E-07	5,16E-08	2,29E-05	4,34E-07
N1563-03	7,08E-08	1,06E-08	2,11E-08	4,59E-09	2,33E-08	2,31E-08	2,12E-07	6,83E-09	1,70E-05	4,09E-07
N1563-04	7,08E-08	1,77E-08	2,11E-08	4,59E-09	2,33E-08	2,31E-08	2,12E-07	6,83E-09	1,70E-05	4,09E-07
N1563-05	6,06E-08	1,73E-08	2,86E-08	7,44E-09	3,22E-08	1,66E-08	2,31E-07	7,85E-08	1,54E-05	2,76E-07
N1563-06	6,06E-08	1,73E-08	2,86E-08	7,44E-09	3,22E-08	1,66E-08	2,31E-07	7,85E-08	1,54E-05	2,76E-07
N1563-07	5,99E-08	1,30E-08	3,32E-08	9,07E-09	2,62E-08	1,63E-08	8,03E-07	1,28E-07	1,94E-05	4,27E-07
N1563-08	5,99E-08	1,30E-08	3,32E-08	9,07E-09	2,62E-08	1,63E-08	8,03E-07	1,28E-07	1,94E-05	4,27E-07
N1563-09	5,99E-08	1,30E-08	3,32E-08	9,07E-09	2,62E-08	1,63E-08	8,03E-07	1,28E-07	1,94E-05	4,27E-07
N1563-10	5,69E-08	9,84E-09	1,80E-08	4,38E-09	5,69E-08	2,33E-08	2,83E-07	1,39E-07	1,58E-05	3,00E-07
N1563-11	5,69E-08	9,84E-09	1,80E-08	4,38E-09	5,69E-08	2,33E-08	2,83E-07	1,39E-07	1,58E-05	3,00E-07

Table S2

Table S2

Sample ID:	OM-1	Lab# N1522-01/N1522-15	J = 0.00039160 ± 0.00000274		
Sanidine		Irradiation #109		reactor	OSIRIS
Flux standard	ACS-2	1.193 Ma	Single crystal total fusion		

N	^{40}Ar (moles)	^{36}Ar V	±% σ_{36}	^{37}Ar V	±%σ_{37}	^{38}Ar V	±%σ_{38}	^{39}Ar V	±%σ_{39}	^{40}Ar V	±%σ_{40}	D[1]	±%σ_D	%^{40}Ar*	Age (ka)	±σ (ka)	K/Ca ± 1σ	
N1522-01	4,555E-15	4,14E-07	5,256	3,26E-05	20,705	7,97E-05	0,206	4,96E-03	0,092	3,32E-03	0,081	1,010	0,07	96,38	456,7	± 1,1	65,3	13,5
N1522-02	1,000E-14	7,29E-06	0,566	3,25E-05	20,705	8,44E-05	0,578	5,61E-03	0,184	7,30E-03	0,151	1,011	0,07	70,22	646,0	± 2,5	74,2	15,4
**N1522-03**	**8,392E-15**	**6,31E-06**	**0,525**	**1,78E-04**	**20,138**	**1,40E-04**	**0,253**	**7,45E-03**	**0,130**	**6,13E-03**	**0,151**	**1,011**	**0,07**	**69,58**	**404,3**	**± 1,5**	**18,0**	**3,6**
N1522-04	1,422E-14	1,52E-05	0,414	3,34E-04	20,080	1,35E-04	0,167	8,32E-03	0,122	1,04E-02	0,120	1,012	0,07	56,50	497,5	± 2,3	10,7	2,1
N1522-05	1,839E-14	1,85E-06	1,938	9,70E-05	20,267	1,06E-04	0,146	7,83E-03	0,165	1,34E-02	0,140	1,013	0,07	95,94	1161,8	± 2,8	34,7	7,0
N1522-06	1,599E-15	1,54E-07	13,552	9,83E-05	20,267	2,89E-05	0,175	1,76E-03	0,148	1,17E-03	0,162	1,009	0,07	97,00	453,3	± 2,8	7,7	1,6
N1522-07	4,652E-15	1,82E-06	1,366	9,81E-05	20,267	6,92E-05	0,289	4,47E-03	0,092	3,40E-03	0,102	1,010	0,07	84,28	451,9	± 1,4	19,6	4,0
N1522-08	3,387E-15	4,52E-07	9,523	9,83E-05	20,267	6,37E-05	0,540	3,66E-03	0,289	2,47E-03	0,153	1,009	0,07	95,00	452,9	± 2,9	16,0	3,2
**N1522-09**	**3,947E-15**	**1,55E-06**	**1,291**	**7,35E-05**	**20,353**	**6,89E-05**	**0,388**	**4,23E-03**	**0,092**	**2,88E-03**	**0,113**	**1,009**	**0,07**	**84,21**	**404,8**	**± 1,2**	**24,8**	**5,0**
N1522-10	3,728E-15	1,03E-06	1,514	7,35E-05	20,353	5,20E-05	0,600	3,72E-03	0,122	2,72E-03	0,175	1,009	0,07	88,98	460,3	± 1,4	21,7	4,4
**N1522-11**	**3,913E-15**	**6,62E-07**	**3,128**	**6,11E-05**	**20,422**	**7,42E-05**	**0,272**	**4,65E-03**	**0,241**	**2,86E-03**	**0,155**	**1,009**	**0,07**	**93,31**	**404,8**	**± 1,5**	**32,8**	**6,7**
N1522-12	5,279E-15	2,78E-06	1,984	6,08E-05	20,422	7,54E-05	0,272	4,65E-03	0,241	3,85E-03	0,153	1,010	0,07	78,63	460,2	± 2,9	32,9	6,7
**N1522-13**	**3,048E-15**	**1,07E-06**	**2,116**	**6,09E-05**	**20,422**	**5,07E-05**	**0,552**	**3,31E-03**	**0,184**	**2,22E-03**	**0,247**	**1,009**	**0,07**	**85,98**	**408,5**	**± 2,0**	**23,4**	**4,8**
N1522-14	3,955E-15	9,57E-07	3,547	4,02E-05	39,733	6,70E-05	0,274	4,09E-03	0,175	2,89E-03	0,102	1,009	0,07	90,25	450,2	± 2,0	43,7	17,4
N1522-15	8,765E-15	7,38E-06	0,613	4,01E-05	39,733	1,05E-04	0,324	6,88E-03	0,114	6,40E-03	0,111	1,011	0,07	65,61	431,3	± 1,7	73,7	29,3

Results	40Ar*/39Ark ± 1σ	Age ± 1σ (Ka)	MSWD	39Ar(k) (n)	K/Ca ± 1σ
Weighted mean	0,5736 ± 0,0011 ± 0,19%	405,2 ± 2,9 ± 0,73%	1,06	1,06 4	22,6 ± 2,8
		Full External Error ± 5,8	1,10	Statistical T ratio	
		Analytical Error ± 0,8	1,0311		

Results	40(a)/36(a) ± 1σ	40(r)/39(k) ± 1σ	Age ± 1σ (ka)	MSWD
[illegible]	296,3217 ± 4,8899 ± 1,65%	0,5745 ± 0,0024 ± 0,42%	405,8 ± 3,3 ± 0,82%	1,47
			Full External Error ± 6,0	
			Analytical Error ± 1,7	

Statistics				
Statistical F ratio	1,05	Convergence	1,486E-03
Error Magnification	1,2142	Number of Iterations	3
Number of Data Points	4	Calculated Line	Weighted York-2

Background corrections OM-1

N	^{36}Ar V	±σ_{36} V	^{37}Ar V	±σ_{37} V	^{38}Ar V	±σ_{38} V	^{39}Ar V	±σ_{39} V	^{40}Ar V	±σ_{40} V
N1522-01	8,29E-08	1,89E-08	1,00E-08	1,00E-09	6,43E-08	1,68E-08	5,55E-07	1,64E-07	2,32E-05	2,90E-07
N1522-02	8,29E-08	1,89E-08	1,00E-08	1,00E-09	6,43E-08	1,68E-08	5,55E-07	1,64E-07	2,32E-05	2,90E-07
N1522-03	6,73E-08	3,84E-10	1,00E-08	1,00E-09	6,43E-08	1,68E-08	5,55E-07	1,64E-07	2,32E-05	2,90E-07
N1522-04	8,29E-08	1,89E-08	1,00E-08	1,00E-09	6,43E-08	1,68E-08	5,55E-07	1,64E-07	2,32E-05	2,90E-07
N1522-05	9,75E-08	1,91E-08	1,00E-08	1,00E-09	8,26E-08	2,69E-08	1,88E-07	4,10E-08	1,37E-05	9,10E-07
N1522-06	9,75E-08	1,91E-08	1,00E-08	1,00E-09	8,26E-08	2,69E-08	1,88E-07	4,10E-08	1,37E-05	9,10E-07
N1522-07	9,57E-08	1,88E-08	1,00E-08	1,00E-09	6,72E-08	2,30E-08	1,03E-06	1,16E-07	2,57E-05	5,97E-07
N1522-08	9,57E-08	1,88E-08	1,00E-08	1,00E-09	6,72E-08	2,30E-08	1,03E-06	1,16E-07	2,57E-05	5,97E-07
N1522-09	9,57E-08	1,88E-08	1,00E-08	1,00E-09	6,72E-08	2,30E-08	1,03E-06	1,16E-07	2,57E-05	5,97E-07
N1522-10	1,07E-07	1,58E-08	1,00E-08	1,00E-09	8,36E-08	2,83E-08	3,88E-06	1,17E-07	3,47E-05	8,62E-07
N1522-11	1,07E-07	1,58E-08	1,00E-08	1,00E-09	8,36E-08	2,83E-08	3,88E-06	1,17E-07	3,47E-05	8,62E-07
N1522-12	1,07E-07	1,58E-08	1,00E-08	1,00E-09	8,36E-08	2,83E-08	3,88E-06	1,17E-07	3,47E-05	8,62E-07
N1522-13	1,07E-07	1,58E-08	1,00E-08	1,00E-09	8,36E-08	2,83E-08	3,88E-06	1,17E-07	3,47E-05	8,62E-07
N1522-14	1,47E-07	2,96E-08	3,17E-08	3,14E-08	7,81E-08	3,47E-08	3,61E-07	1,08E-07	2,29E-05	3,89E-07
N1522-15	1,47E-07	2,96E-08	3,17E-08	3,14E-08	7,81E-08	3,47E-08	3,61E-07	1,08E-07	2,29E-05	3,89E-07

Table S3

Table S3

Sample ID:	PC-6	Lab# N1522-01/N1522-15	J = 0.00038900 ± 0.00000078		
Sanidine		Irradiation #109		reactor	OSIRIS
Flux standard	ACS-2	1.193 Ma	Single crystal total fusion		

N	^{40}Ar (moles)	^{36}Ar V	±% σ_{36}	^{37}Ar V	±%σ_{37}	^{38}Ar V	±%σ_{38}	^{39}Ar V	±%σ_{39}	^{40}Ar V	±%σ_{40}	D[1]	±%σ_D	%^{40}Ar*	Age (ka)	±σ (ka)	K/Ca ± 1σ	
**N1553-01**	**7,042E-15**	**9,90E-08**	**15,460**	**7,95E-05**	**25,519**	**9,03E-05**	**0,462**	**6,03E-03**	**0,122**	**5,14E-03**	**0,091**	**1,010**	**0,07**	**99,60**	**596,0**	**± 1,1**	**32,6**	**± 8,3**
**N1553-02**	**3,550E-15**	**8,53E-08**	**20,016**	**6,98E-05**	**26,365**	**4,45E-05**	**0,416**	**3,05E-03**	**0,130**	**2,59E-03**	**0,081**	**1,009**	**0,07**	**99,32**	**593,0**	**± 1,6**	**18,8**	**± 4,9**
**N1553-03**	**4,182E-15**	**9,83E-08**	**16,766**	**7,97E-05**	**25,519**	**5,23E-05**	**0,324**	**3,57E-03**	**0,139**	**3,05E-03**	**0,071**	**1,010**	**0,07**	**99,33**	**595,4**	**± 1,4**	**19,3**	**± 4,9**
N1553-04	4,165E-15	1,17E-07	15,514	1,39E-04	23,062	5,33E-05	0,466	3,48E-03	0,122	3,04E-03	0,102	1,010	0,07	99,38	610,0	± 1,6	10,7	± 2,5
**N1553-05**	**4,217E-15**	**2,98E-06**	**1,283**	**6,97E-05**	**26,365**	**4,28E-05**	**0,564**	**2,60E-03**	**0,157**	**3,08E-03**	**0,142**	**1,010**	**0,07**	**71,38**	**593,7**	**± 3,5**	**16,0**	**± 4,2**
**N1553-06**	**5,349E-15**	**1,32E-06**	**1,410**	**8,16E-05**	**25,376**	**6,40E-05**	**0,317**	**4,16E-03**	**0,106**	**3,90E-03**	**0,091**	**1,010**	**0,07**	**90,16**	**593,2**	**± 1,4**	**21,9**	**± 5,6**
**N1553-07**	**1,488E-14**	**1,55E-05**	**0,340**	**4,77E-05**	**113,230**	**1,31E-04**	**0,261**	**7,33E-03**	**0,114**	**1,09E-02**	**0,080**	**1,012**	**0,07**	**57,51**	**597,6**	**± 2,2**	**66,1**	**± 74,9**
**N1553-08**	**3,302E-15**	**1,44E-07**	**11,094**	**1,01E-04**	**57,838**	**4,35E-05**	**0,437**	**2,82E-03**	**0,166**	**2,41E-03**	**0,172**	**1,009**	**0,07**	**98,70**	**591,0**	**± 2,5**	**12,0**	**± 6,9**
**N1553-09**	**5,366E-15**	**5,73E-07**	**3,303**	**9,01E-05**	**39,669**	**7,05E-05**	**0,280**	**4,44E-03**	**0,148**	**3,92E-03**	**0,162**	**1,010**	**0,07**	**95,92**	**594,0**	**± 1,7**	**21,2**	**± 8,4**
**N1553-10**	**2,765E-14**	**5,88E-05**	**0,334**	**8,34E-05**	**65,382**	**6,24E-05**	**0,216**	**3,03E-03**	**0,130**	**2,02E-02**	**0,014**	**1,015**	**0,07**	**13,05**	**609,6**	**± 16,7**	**15,6**	**± 10,2**

Results	40Ar*/39Ark ± 1σ	Age ± 1σ (ka)	MSWD	39Ar(k) (n)	K/Ca ± 1σ
		594,6 ± 1,3	1,17		
Weighted mean	0,8474 ± 0,0009 ± 0,10%	594,6 ± 1,1 ± 0,22%	1,13	91,42 9	18,9 ± 2,1
		± 0,22%			
		Full External Error ± 7,4	0,99	Statistical T ratio	
		Analytical Error ± 0,6	1,0616		

Results	40(a)/36(a) ± 1σ	± 1σ	Age ± 1σ (Ka)	MSWD
Inverse Isochron	299,8835 ± 0,8407 ± 0,28%	0,8471 ± 0,0009 ± 0,10%	594,4 ± 1,3 ± 0,22%	1,05
Full External Error			± 7,4	
Analytical Error			± 0,6	

Statistics				
Statistical F ratio	1,12	Convergence	0,0033539273
Error Magnification	1,0232	Number of Iterations	2
Number of Data Points	9	Calculated Line	Weighted York-2

Background corrections PC-6

N	^{36}Ar V	±σ36 V	^{37}Ar V	±σ37 V	^{38}Ar V	±σ38 V	^{39}Ar V	±σ39 V	^{40}Ar V	±σ40 V
N1553-01	8,29E-08	1,12E-08	1,00E-08	2,00E-09	9,11E-08	1,39E-08	3,26E-06	1,40E-07	2,05E-05	2,83E-07
N1553-02	8,29E-08	1,12E-08	1,00E-08	2,00E-09	9,11E-08	1,39E-08	3,26E-06	1,40E-07	2,05E-05	2,83E-07
N1553-03	8,29E-08	1,12E-08	1,00E-08	2,00E-09	9,11E-08	1,39E-08	3,26E-06	1,40E-07	2,05E-05	2,83E-07
N1553-04	6,90E-08	1,14E-08	1,00E-08	2,00E-09	8,47E-08	3,17E-08	1,55E-07	1,28E-07	1,79E-05	4,53E-07
N1553-05	6,90E-08	1,14E-08	1,00E-08	2,00E-09	8,47E-08	3,17E-08	1,55E-07	1,28E-07	1,79E-05	4,53E-07
N1553-06	6,90E-08	1,14E-08	1,00E-08	2,00E-09	8,47E-08	3,17E-08	1,55E-07	1,28E-07	1,79E-05	4,53E-07
N1553-07	7,03E-08	1,24E-08	2,84E-08	2,55E-08	2,41E-08	2,39E-08	2,82E-07	1,00E-07	1,56E-05	3,27E-07
N1553-08	7,03E-08	1,24E-08	2,84E-08	2,55E-08	2,41E-08	2,39E-08	2,82E-07	1,00E-07	1,56E-05	3,27E-07
N1553-09	8,60E-08	1,56E-08	2,11E-08	1,08E-08	2,98E-08	2,54E-08	3,40E-07	1,56E-08	2,60E-05	5,20E-07
N1553-10	8,60E-08	1,56E-08	2,11E-08	1,08E-08	2,98E-08	2,54E-08	3,40E-07	1,56E-08	2,60E-05	5,20E-07

Table S4

Table S4

Sample ID: **PC-5** Lab# N-1552-01/1552-12 J = 0.00038460 ± 0.00000077

Sanidine Irradiation # 109 reactor OSIRIS

Flux standard ACs-2 1.193 Ma Single crystal total fusion

N	^{40}Ar (moles)	^{36}Ar V	±% σ_{36}	^{37}Ar V	±%σ_{37}	^{38}Ar V	±%σ_{38}	^{39}Ar V	±%σ_{39}	^{40}Ar V	±%σ_{40} %	D(1)	±%σ_D	%$^{40}Ar^*$	Age (ka)	±σ (ka)	K/Ca	± 1σ
**N1552-01**	**3,739E-15**	**2,07E-08**	**80,623**	**1,80E-05**	**44,735**	**0,0000499**	**0,290**	**3,07E-03**	**0,193**	**2,73E-03**	**0,202**	**1,00952**	**0,07**	**99,88**	**616,1**	**± 2,1**	**73,1**	**32,7**
N1552-02	3,670E-15	6,08E-07	3,271	5,42E-05	27,509	0,0000283	0,887	2,48E-03	0,166	2,68E-03	0,112	1,00947	0,07	93,40	699,4	± 2,2	19,7	5,4
**N1552-03**	**1,246E-15**	**2,61E-07**	**8,532**	**5,64E-05**	**27,509**	**0,0000154**	**0,819**	**9,35E-04**	**0,203**	**9,09E-04**	**0,175**	**1,00889**	**0,07**	**92,16**	**621,4**	**± 5,4**	**7,1**	**2,0**
**N1552-04**	**2,962E-15**	**1,25E-06**	**1,545**	**5,64E-05**	**27,509**	**0,0000353**	**0,524**	**2,02E-03**	**0,139**	**2,16E-03**	**0,122**	**1,0093**	**0,07**	**83,06**	**617,8**	**± 2,4**	**15,4**	**4,2**
**N1552-05**	**5,303E-15**	**3,07E-06**	**0,724**	**5,63E-05**	**27,509**	**0,0000583**	**0,511**	**3,33E-03**	**0,086**	**3,87E-03**	**0,111**	**1,00987**	**0,07**	**76,52**	**617,1**	**± 1,8**	**25,4**	**7,0**
N1552-06	3,446E-15	6,94E-07	2,980	5,97E-05	27,510	0,0000286	0,422	1,98E-03	0,130	2,52E-03	0,125	1,00944	0,07	92,03	812,0	± 2,7	14,3	3,9
**N1552-08**	**1,549E-15**	**4,91E-07**	**4,271**	**5,99E-05**	**27,510**	**0,0000197**	**0,537**	**1,10E-03**	**0,157**	**1,13E-03**	**0,176**	**1,00896**	**0,07**	**87,68**	**625,7**	**± 4,4**	**7,9**	**2,2**
N1552-09	2,247E-15	5,07E-07	4,175	5,99E-05	27,510	0,0000174	0,821	1,59E-03	0,139	1,64E-03	0,162	1,00911	0,07	91,12	650,3	± 3,2	11,5	3,2
N1552-10	1,539E-15	6,95E-07	4,250	5,73E-05	31,626	0,0000122	0,739	9,07E-04	0,122	1,12E-03	0,176	1,00896	0,07	82,08	705,1	± 7,1	6,8	2,2
N1552-11	2,772E-15	1,52E-06	1,468	4,39E-05	55,197	0,0000238	0,620	1,34E-03	0,130	2,02E-03	0,163	1,00926	0,07	77,82	814,0	± 4,3	13,2	7,3
N1552-12	2,372E-15	5,34E-08	32,420	4,19E-05	80,615	0,0000317	0,380	1,69E-03	0,166	1,73E-03	0,124	1,00916	0,07	99,43	706,9	± 3,0	17,3	14,0

Results	40Ar*/39Ark ± 1σ	Age ± 1σ (ka)	MSWD	39Ar(k) (n)	K/Ca ± 1σ
Weighted mean	0,8904 ± 0,0017 ± 0,19%	617,7 ± 1,7 ± 0,28%	1,13	5	9,1 ± 2,4
		Full External Error ± 7,8	1,06	Statistical T ratio	
		Analytical Error ± 1,2	1,0611		

Results	40(a)/36(a) ± 1σ	± 1σ	Age ± 1σ (ka)	MSWD
Inverse Isochron	299,4177 ± 5,2028 ± 1,74%	0,8899 ± 0,0034 ± 0,38%	617,4 ± 2,6 ± 0,43%	1,50
Full External Error			± 8,0	
Analytical Error			± 2,3	

Statistics			
Statistical F ratio	1,09	Convergence	0,0021677216
Error Magnification	1,2228	Number of Iterations	2
Number of Data Points	5	Calculated Line	Weighted York-2

Background corrections PC-5

N	^{36}Ar V	±σ36 V	^{37}Ar V	±σ37 V	^{38}Ar V	±σ38 V	^{39}Ar V	±σ39 V	^{40}Ar V	±σ40 V
N1552-01	6,95E-08	1,26E-08	1,00E-08	2,00E-09	5,71E-08	2,18E-08	4,52E-07	1,43E-07	1,96E-05	3,61E-07
N1552-02	9,78E-08	8,95E-09	1,00E-08	2,00E-09	5,93E-08	2,55E-08	8,92E-07	1,01E-07	1,93E-05	4,56E-07
N1552-03	5,48E-08	1,44E-08	1,00E-08	2,00E-09	6,49E-08	2,28E-08	1,61E-07	8,50E-08	1,70E-05	2,55E-07
N1552-04	5,48E-08	1,44E-08	1,00E-08	2,00E-09	6,49E-08	2,28E-08	1,61E-07	8,50E-08	1,70E-05	2,55E-07
N1552-05	7,57E-08	1,57E-08	1,00E-08	2,00E-09	2,32E-08	2,02E-08	3,84E-07	1,08E-07	1,84E-05	5,52E-07
N1552-06	3,93E-07	1,65E-08	1,00E-08	2,00E-09	1,01E-07	4,17E-08	6,06E-08	4,81E-08	9,36E-05	1,78E-07
N1552-08	6,92E-08	1,49E-08	1,00E-08	2,00E-09	9,30E-08	1,60E-08	3,46E-07	4,40E-08	1,86E-05	1,56E-07
N1552-09	6,92E-08	1,49E-08	1,00E-08	2,00E-09	9,30E-08	1,60E-08	3,46E-07	4,40E-08	1,86E-05	1,56E-07
N1552-10	1,33E-07	1,85E-08	1,00E-08	2,90E-09	8,19E-08	2,16E-08	1,27E-07	9,00E-08	2,89E-05	2,89E-07
N1552-11	1,20E-07	1,43E-08	3,00E-08	6,00E-09	2,97E-08	1,47E-08	3,24E-07	8,09E-08	2,84E-05	3,70E-07
N1552-12	1,20E-07	1,43E-08	3,00E-08	6,00E-09	2,97E-08	1,47E-08	3,24E-07	8,09E-08	2,84E-05	3,70E-07

Table S5

Table S5

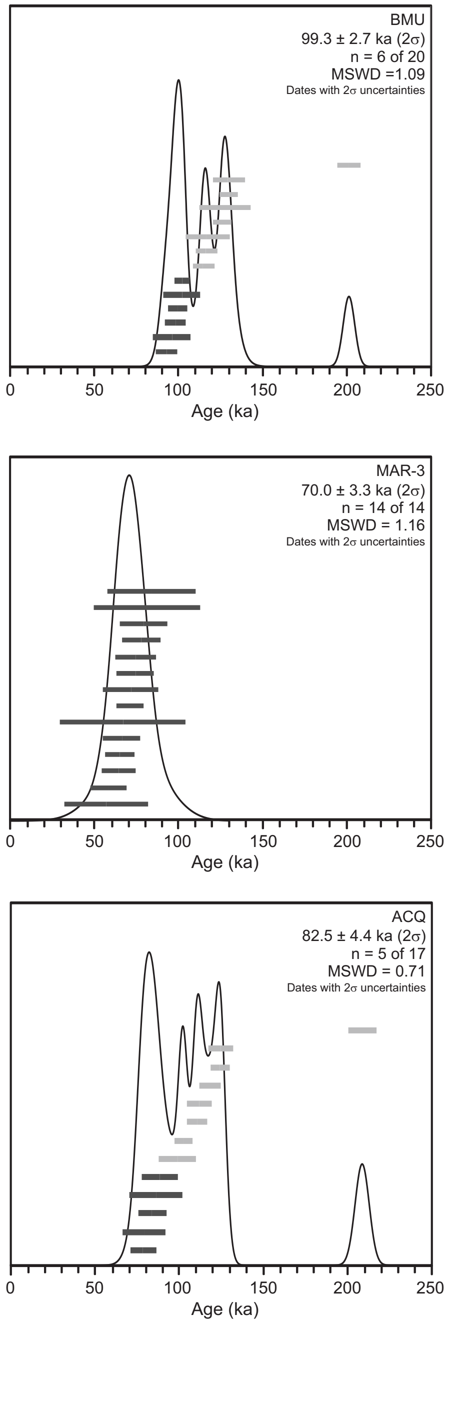
BMU
99.3 ± 2.7 ka (2σ)
n = 6 of 20
MSWD =1.09
Dates with 2σ uncertainties
0 50 100 150 200 250
Age (ka)
MAR-3
70.0 ± 3.3 ka (2σ)
n = 14 of 14
MSWD = 1.16
Dates with 2σ uncertainties
0 50 100 150 200 250
Age (ka)
ACQ
82.5 ± 4.4 ka (2σ)
n = 5 of 17
MSWD = 0.71
Dates with 2σ uncertainties
0 50 100 150 200 250
Age (ka)

Figure S3

Table S6

$^{40}Ar/^{39}Ar$ results

Single crystal fusions

Sample: BMU   J-value: 0,0003856 ± 0,0000006 (2σ)
Material: sanidine

File	Laser power (%)	$^{40}Ar \pm 2\sigma_{40}$ (cps) (cps)	$^{39}Ar \pm 2\sigma_{39}$ (cps) (cps)	$^{37}Ar \pm 2\sigma_{37}$ (cps) (cps)	$^{36}Ar \pm 2\sigma_{36}$ (cps) (cps)	$^{40}Ar^*/^{39}Ar_K \pm 2\sigma$	$\%^{40}Ar^*$	Age (ka) ± 2σ (ka)	K/Ca	Included in wtd mean
NAH0228	30	25986 ± 46	117595 ± 335	1223 ± 214	15,87 ± 2,18	0,180966 ± 0,021529	81,89	127,7 ± 15,2	41,339	
NAH0231	30	183467 ± 122	412265 ± 632	4821 ± 294	413,98 ± 5,97	0,145605 ± 0,007228	32,72	102,8 ± 5,1	36,773	YES
NAH0233	30	262180 ± 146	362713 ± 536	3402 ± 221	679,99 ± 10,03	0,163312 ± 0,008987	22,59	115,3 ± 6,3	45,842	
NAH0236	30	251908 ± 143	280211 ± 406	3679 ± 240	236,68 ± 5,27	0,647320 ± 0,006248	72,00	456,8 ± 4,4	32,750	
NAH0238	30	193461 ± 121	309239 ± 465	2187 ± 248	462,81 ± 6,95	0,178793 ± 0,007861	28,58	126,2 ± 5,5	60,814	
NAH0241	30	115772 ± 96	151608 ± 262	1746 ± 256	243,03 ± 4,52	0,285405 ± 0,009703	37,37	201,4 ± 6,8	37,334	
NAH0243	30	301888 ± 142	362135 ± 509	3046 ± 230	811,46 ± 10,07	0,164754 ± 0,008830	19,76	116,3 ± 6,2	51,122	
NAH0246	30	61614 ± 80	258717 ± 578	3131 ± 285	81,53 ± 3,31	0,144479 ± 0,015796	60,67	102,0 ± 11,1	35,535	YES
NAH0248	30	228165 ± 147	320662 ± 506	2547 ± 252	321,75 ± 5,51	0,412056 ± 0,006363	57,91	290,8 ± 4,5	54,146	
NAH0251	30	185522 ± 136	266058 ± 448	2365 ± 250	458,27 ± 6,69	0,183211 ± 0,008570	26,27	129,3 ± 6,0	48,384	
NAH0253	30	243372 ± 136	183529 ± 288	2483 ± 219	349,84 ± 6,37	0,757501 ± 0,010595	57,12	534,6 ± 7,5	31,781	
NAH0256	30	140948 ± 93	371967 ± 587	4552 ± 265	296,68 ± 5,63	0,141226 ± 0,008340	37,27	99,7 ± 5,9	35,136	YES
NAH0258	30	45765 ± 63	73894 ± 176	862 ± 230	112,16 ± 4,27	0,166534 ± 0,018374	26,89	117,5 ± 13,0	36,864	
NAH0261	30	202917 ± 140	381342 ± 591	3192 ± 309	141,30 ± 4,55	0,421610 ± 0,006039	79,23	297,6 ± 4,3	51,375	
NAH0263	30	43643 ± 60	83679 ± 196	495 ± 210	32,86 ± 3,19	0,404242 ± 0,013544	77,51	285,3 ± 9,6	72,680	
NAH0266	30	179575 ± 109	636583 ± 904	7370 ± 277	305,09 ± 5,15	0,139382 ± 0,008787	49,41	98,4 ± 6,2	37,139	YES
NAH0268	30	108089 ± 90	184703 ± 323	2541 ± 256	280,90 ± 5,64	0,131704 ± 0,010427	22,51	93,0 ± 7,4	31,258	YES
NAH0271	30	368970 ± 177	321005 ± 454	3299 ± 279	380,32 ± 6,52	0,795969 ± 0,006437	69,25	561,7 ± 4,5	41,842	
NAH0273	30	241647 ± 141	180523 ± 276	1886 ± 293	727,15 ± 9,39	0,136276 ± 0,015757	10,18	96,2 ± 11,1	41,154	YES
NAH0276	30	130491 ± 106	142283 ± 249	1625 ± 260	349,43 ± 6,28	0,184271 ± 0,013617	20,09	130,1 ± 9,6	37,659	
						weighted mean age (6 of 20):		99,3 ± 2,7		

Sample: MAR-3   J-value: 0,0003856 ± 0,0000006 (2σ)
Material: sanidine

File	Laser power (%)	$^{40}Ar \pm 2\sigma_{40}$ (cps) (cps)	$^{39}Ar \pm 2\sigma_{39}$ (cps) (cps)	$^{37}Ar \pm 2\sigma_{37}$ (cps) (cps)	$^{36}Ar \pm 2\sigma_{36}$ (cps) (cps)	$^{40}Ar^*/^{39}Ar_K \pm 2\sigma$	$\%^{40}Ar^*$	Age (ka) ± 2σ (ka)	K/Ca	Included in wtd mean
NAH0280	30	243719 ± 112	58965 ± 99	877 ± 227	797,55 ± 10,33	0,095664 ± 0,052773	2,31	67,5 ± 37,2	28,917	YES
NAH0286	30	54326 ± 82	160083 ± 397	1716 ± 284	132,87 ± 4,41	0,091866 ± 0,014424	27,07	64,8 ± 10,2	40,117	YES
NAH0289	30	18497 ± 64	43609 ± 224	488 ± 210	50,12 ± 4,31	0,081375 ± 0,035316	19,18	57,4 ± 24,9	38,453	YES
NAH0291	30	54247 ± 77	63006 ± 158	812 ± 204	160,24 ± 4,85	0,102156 ± 0,023531	11,86	72,1 ± 16,6	33,382	YES
NAH0294	30	58065 ± 85	105309 ± 261	1298 ± 310	161,19 ± 4,87	0,094842 ± 0,015670	17,20	66,9 ± 11,1	34,888	YES
NAH0301	30	145368 ± 122	126572 ± 224	1200 ± 253	441,94 ± 6,97	0,106255 ± 0,016739	9,25	75,0 ± 11,8	45,368	YES
NAH0306	30	48868 ± 72	100250 ± 251	858 ± 233	128,17 ± 4,51	0,105896 ± 0,015843	21,72	74,7 ± 11,2	50,242	YES
NAH0309	30	28056 ± 62	70895 ± 245	1137 ± 254	67,36 ± 3,45	0,112817 ± 0,020249	28,51	79,6 ± 14,3	26,804	YES
NAH0311	30	114344 ± 103	115448 ± 211	1197 ± 280	350,88 ± 5,79	0,083299 ± 0,015371	8,41	58,8 ± 10,8	41,471	YES
NAH0314	30	17704 ± 53	110116 ± 485	1412 ± 255	16,72 ± 2,82	0,115909 ± 0,044375	72,09	81,8 ± 31,3	33,525	YES
NAH0321	30	109968 ± 94	156342 ± 287	1081 ± 285	315,32 ± 5,54	0,101233 ± 0,011503	14,39	71,5 ± 8,1	62,198	YES
NAH0324	30	139934 ± 90	169099 ± 280	1612 ± 202	416,27 ± 6,74	0,092774 ± 0,012427	11,21	65,5 ± 8,8	45,116	YES
NAH0326	30	15818 ± 49	74510 ± 348	1035 ± 284	23,16 ± 2,87	0,120035 ± 0,037041	56,54	84,7 ± 26,1	30,959	YES
NAH0329	30	35910 ± 58	97964 ± 267	1268 ± 251	84,10 ± 3,50	0,110752 ± 0,016172	30,21	78,2 ± 11,4	33,221	YES
						weighted mean age (14 of 14):		70,0 ± 3,3		

Sample: ACQ   J-value: 0,0003856 ± 0,0000006 (2σ)
Material: sanidine

File	Laser power (%)	$^{40}Ar \pm 2\sigma_{40}$ (cps) (cps)	$^{39}Ar \pm 2\sigma_{39}$ (cps) (cps)	$^{37}Ar \pm 2\sigma_{37}$ (cps) (cps)	$^{36}Ar \pm 2\sigma_{36}$ (cps) (cps)	$^{40}Ar^*/^{39}Ar_K \pm 2\sigma$	$\%^{40}Ar^*$	Age (ka) ± 2σ (ka)	K/Ca	Included in wtd mean
NAH0331	30	120064 ± 105	146014 ± 269	2020 ± 294	92,89 ± 4,07	0,632899 ± 0,009133	76,97	446,7 ± 6,4	31,082	
NAH0334	30	137817 ± 114	371749 ± 637	2934 ± 246	253,46 ± 5,79	0,167251 ± 0,008989	45,11	118,0 ± 6,3	54,478	
NAH0336	30	190510 ± 118	144756 ± 232	1847 ± 255	584,05 ± 8,54	0,111953 ± 0,017837	8,51	79,0 ± 12,6	33,702	YES
NAH0339	30	166283 ± 116	211179 ± 336	3116 ± 305	432,50 ± 6,98	0,176578 ± 0,010480	22,43	124,6 ± 7,4	29,143	
NAH0344	30	316680 ± 160	491098 ± 686	6499 ± 321	822,64 ± 10,87	0,145227 ± 0,007592	22,52	102,5 ± 5,4	32,495	
NAH0346	30	189445 ± 134	278841 ± 440	2190 ± 292	258,46 ± 5,69	0,402742 ± 0,007241	59,28	284,2 ± 5,1	54,747	
NAH0349	30	273697 ± 144	322439 ± 456	4865 ± 317	748,22 ± 8,86	0,156680 ± 0,008733	18,46	110,6 ± 6,2	28,499	
NAH0354	30	214228 ± 111	203086 ± 294	2681 ± 290	516,54 ± 7,89	0,296004 ± 0,011889	28,06	208,9 ± 8,4	32,570	
NAH0356	30	111350 ± 96	597037 ± 1009	7185 ± 303	123,55 ± 4,09	0,125133 ± 0,015142	67,09	88,3 ± 10,7	35,732	YES
NAH0359	30	161498 ± 94	212846 ± 317	2223 ± 231	427,95 ± 6,88	0,158760 ± 0,010241	20,92	112,1 ± 7,2	41,163	
NAH0361	30	172441 ± 118	320565 ± 507	3186 ± 249	388,94 ± 7,07	0,175938 ± 0,008247	32,71	124,2 ± 5,8	43,266	
NAH0364	30	65342 ± 75	172680 ± 362	1484 ± 239	150,14 ± 4,23	0,118959 ± 0,011695	31,44	84,0 ± 8,3	50,025	YES
NAH0369	30	107988 ± 92	178770 ± 314	2262 ± 320	138,01 ± 4,30	0,374039 ± 0,008668	61,92	264,0 ± 6,1	33,979	
NAH0371	30	70180 ± 84	93059 ± 202	1291 ± 294	191,49 ± 4,64	0,140347 ± 0,015684	18,61	99,1 ± 11,1	30,998	
NAH0374	30	132178 ± 104	120801 ± 213	2502 ± 242	145,13 ± 4,04	0,736607 ± 0,010354	67,32	519,9 ± 7,3	20,757	
NAH0376	30	109830 ± 106	165396 ± 303	2026 ± 233	306,43 ± 5,54	0,111325 ± 0,011037	16,76	78,6 ± 7,8	35,096	YES
NAH0380	30	36048 ± 57	194170 ± 493	2443 ± 259	41,60 ± 2,94	0,122145 ± 0,022561	65,79	86,2 ± 15,9	34,178	YES
						weighted mean age (5 of 17):		82,5 ± 4,4		

The values in this table have been corrected for instrument background, source mass bias, detector efficiency, and decay of 37Ar and 39Ar

Instrument: Noblesse 5-collector mass spectrometer
Standard: Alder Creek rhyolite sanidine
Standard age (Ma): 1,1864 ± 0,0003   Jicha et al. (2016)

Atmospheric argon ratios		
$^{40}Ar/^{36}Ar$	298,56 ± 0,31	Lee et al. (2006)
$^{38}Ar/^{36}Ar$	0,1885 ± 0,0003	Lee et al. (2006)

Interfering isotope production ratios		
$(^{40}Ar/^{39}Ar)_K$	0,00054 ± 0,00014	Jicha & Brown (2014)
$(^{38}Ar/^{39}Ar)_K$	0,01210 ± 0,00002	Jicha & Brown (2014)
$(^{39}Ar/^{37}Ar)_{Ca}$	0,000695 ± 0,00001	Renne et al. (2013)
$(^{38}Ar/^{37}Ar)_{Ca}$	0,0000196 ± 0,0000008	Renne et al. (2013)
$(^{36}Ar/^{37}Ar)_{Ca}$	0,000265 ± 0,00002	Renne et al. (2013)

Decay constants		
λ_{40Ar}	$(0.580 \pm 0.014) \times 10^{-10}\ a^{-1}$	Min et al. (2000)
λ_{B^-}	$(4.884 \pm 0.099) \times 10^{-10}\ a^{-1}$	Min et al. (2000)
^{39}Ar	$(2.58 \pm 0.03) \times 10^{-3}\ a^{-1}$	Stoenner et al. (1965)
^{37}Ar	$(8.23 \pm 0.042) \times 10^{-4}\ h^{-1}$	Stoenner et al. (1965)
^{36}Cl	$(2.303 \pm 0.046) \times 10^{-6}\ a^{-1}$	
