# Supplementary material for: Volcano-tectonic deformation in the Monti Sabatini Volcanic District at the gates of Rome (central Italy): evidence from new geochronologic constraints on the Tiber River MIS 5 terraces
Source: Sci Rep. 2019 Aug 8;9:11496. doi: 10.1038/s41598-019-47585-8 (PMC6687886; doi:10.1038/s41598-019-47585-8)
Supplement: Supplementary file 2 — Supplementary Material 2 [file 41598_2019_47585_MOESM2_ESM.pdf]

Volcano-tectonic deformation in the Monti Sabatini Volcanic District at the gates of Rome (central Italy): evidence from new geochronologic constraints on the Tiber River MIS 5 terraces

Marra, F.[1*], Florindo, F.[1], Jicha, B.[2], Nomade, S.[3], Palladino, D.M.[4], Pereira, A.[5,6], Sottili, G.[4], Tolomei, C.[1]

1) Istituto Nazionale di Geofisica e Vulcanologia, Via di Vigna Murata 605, 00143 Rome, Italy
2) Department of Geoscience, University of Wisconsin-Madison, USA
3) Laboratoire des Sciences du Climat et de l'Environnement, LSCE/IPSL, CEA-CNRS-UVSQ, Université Paris-Saclay, F-91191 Gif-sur-Yvette, France
4) Dipartimento di Scienze della Terra, "Sapienza" Università di Roma, Piazzale Aldo Moro 5, 00185 Roma, Italy
5) UMR 7194 HNHP MNHN-CNRS-UPVD, Départ. Homme et Environ. du MNHN,1 rue René Panhard, 75013
6) Ecole française de Rome, Piazza Farnese, IT-00186, Roma, Italy

*Corresponding author: fabrizio.marra@ingv.it

Supplementary Material #2 - Detailed stratigraphy of the investigated sites and supplementary figures

Passo Corese

A continuous succession of volcanic deposits, mainly air-fall, occurs above the sedimentary deposits of the MIS 15 aggradational succession, at the top of the hill at Passo Corese. Four white pumice layers, 10 to 50 cm thick, are identified (pu1-4, Figure 2a) and correlated with the First ashfall Deposits (FAD) of the MSVD [1, 2]. The FAD deposits are characterized by dominantly pyroclastic-flow units made up of massive or stratified ashy-pumiceous layers with abundant sedimentary lithics from the Morlupo-Castelnuovo di Porto eruptive center interbedded with pumice fall deposits and palaeosoils. In proximal settings, individual pumice layers, with poorly vesiculated, cm-sized pumice juvenile clasts and abundant lava lithics, display maximum thicknesses of 1.5-2 m.
The $^{40}Ar/^{39}Ar$ age of 591.3 ± 2.6 ka yielded by the lowest pumice layer, as well as its depositional and compositional features, allows unambiguous correlation with the FAD 1 pumice layer, part of the Tufo Giallo di Castelnuovo di Porto (TGCP) Eruption Cycle (589 ± 4 ka; [2] of the (MSVD). Consistent with this correlation, a discontinuous, up to 15 cm thick, ash bed underlying the FAD 1 pumice layer (Suppl. Figure 2a-a') shows the typical fine-grained, pale yellow aspect of the TGCP main pyroclastic flow unit. The three overlying pumice layers are separated by pedogenized ash deposits and correlate well with the basal fallout horizons of the three Plinian eruptions occurred at the MSVD at 558±14 ka (FAD 2), 546 ± 5 ka (FAD 3), and 516 ± 1 ka (Tufo Giallo di Prima Porta -TGPP), respectively [2, 3].
Upsection, the occurrence of the Tufo Rosso a Scorie Nere of the MSVD (STRSN; 452±2 ka, [1], a pyroclastic flow deposit up to several tens of meters thick in proximal settings, characterized by a reddish and welded matrix containing the idiosyncratic black vitreous, sanidine-bearing *fiammae* and thermometamorphosed lithic clasts (Suppl. Figure 2b-b'), further supports the above correlation. The STRSN pyroclastic-flow deposit fills a marked paleoincision, consistent with its emplacement during the MIS 14 sea-level fall. Finally, it is overlain by a compositionally zoned fallout deposit (Fall E; Suppl. Figure 2c-c') that represents a stratigraphic marker on a regional scale [2]. Indeed, the Fall E pumice fallout, with a maximum thickness of ~1 m recorded in the Monti Sabatini southern sector, is emplaced on top of an immature paleosoil developed on the Tufo Rosso a Scorie Nere pyroclastic flow deposit and it is made up of whitish, moderately vesiculated pumice clasts and rare lava and sedimentary lithic clasts.
The overall sedimentary features and chrono-stratigraphic relationships of the two aggradational successions exposed at Passo Corese mirror those characterizing their coastal equivalent at Ponte Galeria, represented by the Santa Cecilia and the Ponte Galeria 2 Formations ([4]; Suppl. Figure #2-2b).
The oldest volcanic layer (PC-5) within the upper sandy clay succession (Figure 2) is dated at 614.3 ±3.4 ka, and its deposition occurred in the late stages of sea-level rise during MIS 15.5. The age and stratigraphic position of this layer are indeed equivalent to those of the 614 ± 3 ka tephra occurring in the lagoon deposits (Cerastoderma-bearing clay) of the Santa Cecilia Formation at Ponte Galeria ([4]; Suppl. Figure #2-2b). In

addition, the age of 591.3 ± 2.6 ka yielded by sample PC-6 provides an upper constraint on the completion of glacial termination VII for the deposition of this sedimentary succession.
Similar to the Santa Cecilia Formation at Ponte Galeria, only the upper, fine-grained portion of the MIS 15 aggradational succession is exposed at Passo Corese. The basal coarse portion (gravel) is supposed to occur at lower elevation, at the base (not exposed) of the incision originated by erosion during the regressive phase culminating during sea-level lowstand of MIS 16, which provides an unconformable contact with the underlying aggradational succession correlated with MIS 17. In contrast, this older aggradational succession correlating with MIS 17 is almost entirely exposed. Also similar to the stratigraphic setting at Ponte Galeria, the top of the MIS 17 aggradational succession at Passo Corese occurs a few meters below that of the MIS 15 aggradational succession (see Figure 2a). The thickened basal coarse portion, made up of repeated alternations of gravel and clay layers (Figure S2a), mirrors that characterizing the PG 2 Formation at Ponte Galeria (Suppl. Figure #2-2b), where this feature has been interpreted as the result of syn-sedimentary tectonic lowering of the base level during the time span encompassing glacial terminations VIIIa and VIIIb ([4, 5]; Suppl. Figure #2-2b). At Passo Corese, enduring tectonic subsidence may be regarded as the cause of the higher elevation gain between the top surface of the MIS 15 aggradational succession and that of MIS 17.

Ponte Sfondato

An almost complete exposure of the volcanic succession is observable in an ancient quarry cut at the top of the hilly area, between 110 and 90 m a.s.l. (Suppl. Figure 3b). Here, the STRSN pyroclastic-flow deposit displays an erosional basal contact with a thick succession of fallout deposits and subordinate pyroclastic-flow deposits. The occurrence of the characteristic stratigraphic marker represented by the Holocrystalline Lithic Layer of the Grottarossa Pyroclastic Sequence [1, 3] below the fallout deposits (Suppl. Figure 3b), provides correlation with the Tufi Terrosi con Pomici Bianche succession [1, 6], dated 498 ± 2 - 461 ± 2 ka [3]. This widespread lithic-rich breccia on top of the Grottarossa Pyroclastic Sequence deposits, up to ~1 m thick, contains dominantly holocrystalline lithic clasts with mm-sized clinopyroxene and dark mica, and subordinate lava lithic clasts. Juvenile scoria clasts, up to cm-sized, are poorly vesicular and porphyritic with mm-sized crystals of leucite. Further downsection, a partially re-mobilized pumice fall deposit occurs within a paleoincision, below this stratigraphic marker. Lithostratigraphic and geomorphologic features allow to correlate the pumice layer with the fallout deposit of the Tufo Giallo di Prima Porta (TGPP, [1], emplaced 516 ± 1 ka during the erosional phase triggered by the sea-level fall of MIS 13.2 [3]. This Plinian-style fallout deposit, made up of inversely graded, highly vesicular white pumice occurs at the base of the Tufo Giallo di Prima Porta (TGPP) pyroclastic flow deposit in the North-Eastern sector of the Sabatini Volcanic District, where it reaches a maximum thickness of 2.5 m (Morlupo-Castelnuovo di Porto vent area; [2]). The TGPP basal fallout lies on top of a mature, dark brown paleosoil; pumice clasts present euhedral sanidine (2 mm-sized) in the phenocrysts assemblage, while sanidine and clinopyroxene are frequent in the groundmass.

Cretone Basin

Based on geochronologic and biochronologic age constraints from pyroclastic layers and vertebrate fossil remains intercalated in the lacustrine succession, [7] correlated each terrace with a sea-level highstand, encompassing MIS 15 through MIS 5, following the basic principle of a staircase geometry (Suppl. Figure 4). Later on, [8] used the correlations assessed at Cretone as a reference to pinpoint the elevations of the fluvial terraces reconstructed through geomorphologic analysis in the wider sector of the Tiber River Valley north of Rome. Based on the occurrence of lacustrine deposits geochronologically constrained within MIS 15 at an elevation up to 110 m a.s.l. at the Osteria Moricone site in the Cretone Basin (Suppl. Figure 4), [8] correlated with MIS 15 the paleo-surface defined by the class of topographic culminations ranging 101-110 m a.s.l. in the Tiber River Valley.
[7] hypothesized that around 600 ka, during an intensive phase of extensional tectonics at the onset of the large explosive activity of the Latium volcanoes, the combined effect of uplift and fault displacement gave birth to the main, lower lacustrine basin. Contemporaneously, a minor, upper lacustrine basin originated in consequence of fault damming at Osteria Moricone (see evolutionary scheme in Suppl. Figure 4c-c''). The upper lacustrine basin remained isolated and was not affected by the base level change induced by glacio-eustacy in the valley of the Tiber River, enabling almost continuous sedimentation. By contrast, in the lower basin, discontinuously emplaced fluvial-lacustrine successions were progressively dislocated by uplift and continued faulting (Suppl. Figure 4c'-c'').

Monte Maggiore

Remains of *Ursus* sp., *Elephas* sp., *Elephas (Palaeoloxodon) antiquus, Equus caballus, Equus caballus* cfr. *steinheimensis, Equus hydruntinus, Dicerorhinus* cfr. *hemitoecus, Sus scrofa, Dama dama, Cervus elaphus and Bos primigenius* recovered from a gravel layer in this quarry were described by [9], who attributed the sedimentary deposits hosting the fossils to the Aurelia Formation (MIS 9). However, based on our redetermination of the fallow deer as *Dama dama tiberina*, which in the Italian peninsula has its First Occurrence in MIS 8.5 and is replaced by the sub-species *Dama dama dama* in MIS 5.5 [10, and references therein], we correlate instead the lower aggradational succession including the gravel bed in which the fossils were recovered to the Vitinia Formation and MIS 7, providing a post-quem age of 200 ka for the 56 m terrace in this location (Suppl. Figure 5). Indeed, the fallow deer remain of Monte Maggiore, even if present only with a metatarsal, can be regarded as *D. d. tiberina* based on the measure of the proximal antero-posterior diameter reported in [9]: 28.0 against values ranging 19.0-25.0 for *Dama dama dama* [11].
Moreover, stratigraphic scheme provided in [9] shows the occurrence of two aggradational successions, as evidenced by:
i- a lower, bi-parted horizon of gravels in which the fossil providing correlation with MIS 7 occurred (G-1A and G-1B in Suppl. Figure 5), overlain by a grading upward sandy-clay deposit with freshwater mollusks (S1 in Suppl. Figure 5), and by
ii- an upper gravel horizon (G-2 in in Suppl. Figure 5), passing upward to coarse sand (S2 in Suppl. Figure 5). This second aggradational deposit underlies the terraced surface culminating 56 m a.s.l. which consequently must be ascribed to the following glacio-eustatic cycle of MIS 5.5. Consistently, this elevation corresponds to the MIS 5 paleo-surface reconstructed in this area by [7], and to MIS 5.5 in the present work.

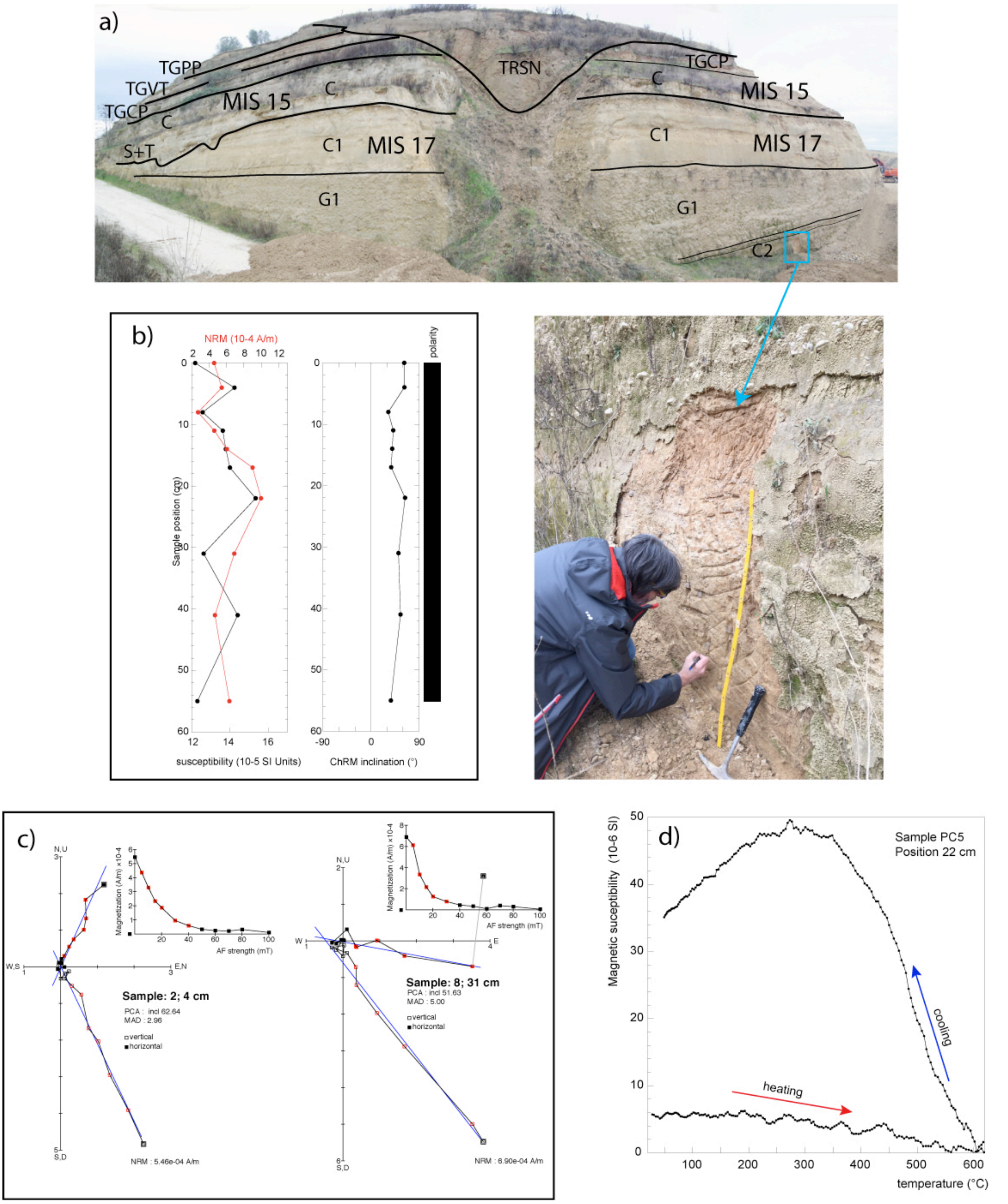

Figure S2-1 - a) Photograph of north-western face of Passo Corese quarry (see location in Figure 2c) showing the outcropping sedimentary and volcanic deposits, and the sampled clay section for paleomagnetic investigations; Legend: TGCP: Tufo Giallo di Castelnuovo di Porto (589±4 ka), TGVT: Tufo Giallo della Via Tiberina (546±5 ka), TGPP: Tufo Giallo di Prima Porta (516±1 ka), TRSN: Tufo Rosso a Scorie Nere (552±2 ka), C: clay, G: gravel, S: sand, T: travertine. b) Stratigraphic variation of NRM, magnetic susceptibility, ChRM inclinations and magnetic polarity zonation. PCA= Principal Component Analysis; MAD= Maximum Angular Deviation. c) Vector component diagrams of demagnetization behavior and intensity decay plots of two representative samples. Photos by authors.

d) Temperature dependence of low-field magnetic susceptibility showing a Curie temperature of ca. 580°C suggesting the presence of Magnetite. The cooling curve (blu arrow) have higher k than the heating curve (red arrow) indicating production of new magnetic phases during the heating cycle. The data are corrected for the diamagnetism of the oven.

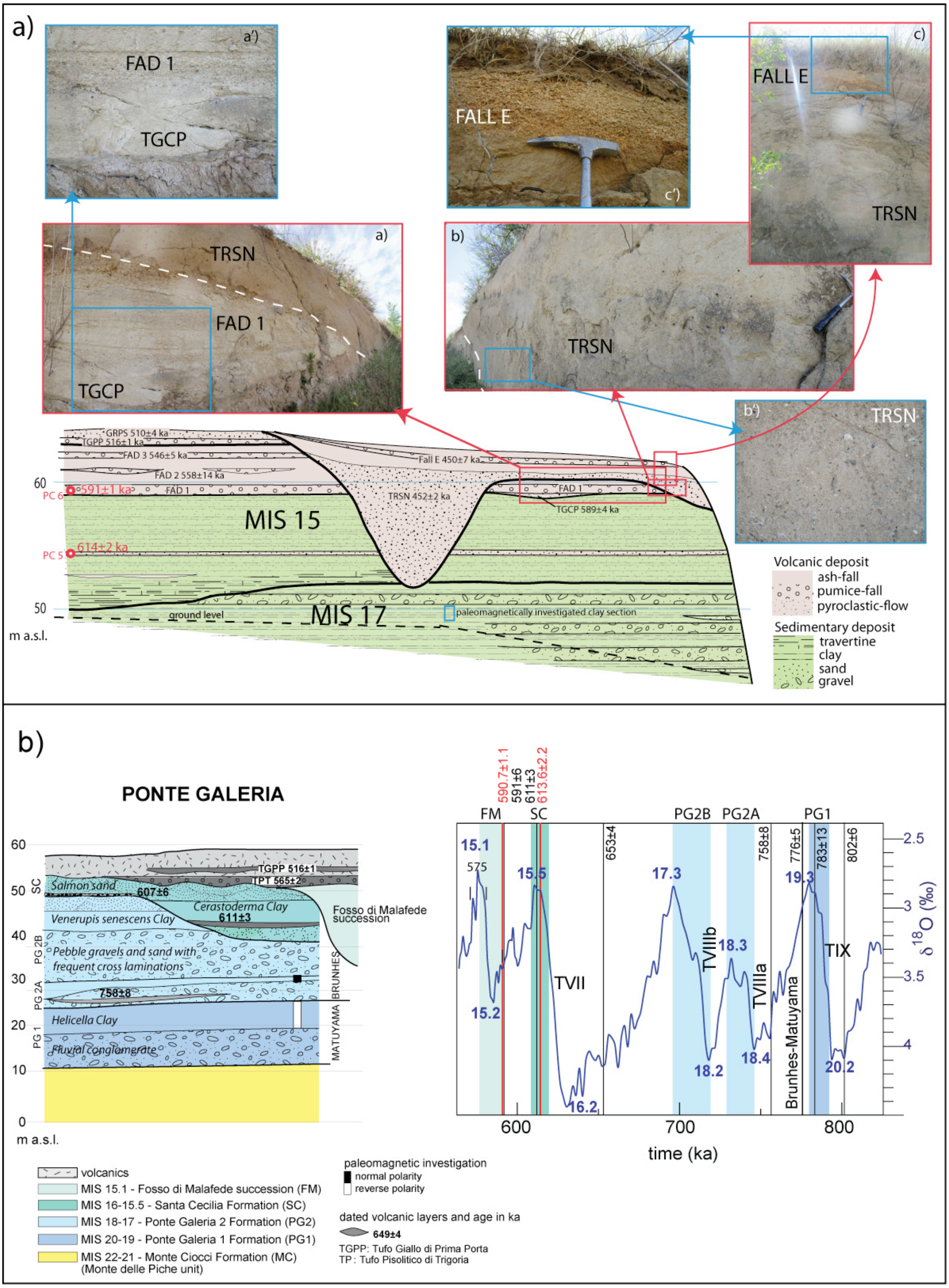

Figure S2-2 - a) Detail on the stratigraphy of the volcanic deposits cropping out in Passo Corese. Legend: TGCP: Tufo Giallo di Castelnuovo di Porto, TGPP: Tufo Giallo di Prima Porta, GRPS: Grottarossa Pyroclastic Sequence, TRSN: Tufo Rosso a Scorie Nere. b) Stratigraphic scheme showing the aggradational successions of MIS 19 through MIS 15 in the coastal sector of Rome (Ponte Galeria area) The geochrnonologic and paleomagnetic constraints providing correlation with the Oxygen isotopes curve are shown, along with those (red vertical bars) from the equivalent successions investigated in Passo Corese. Photos by authors.

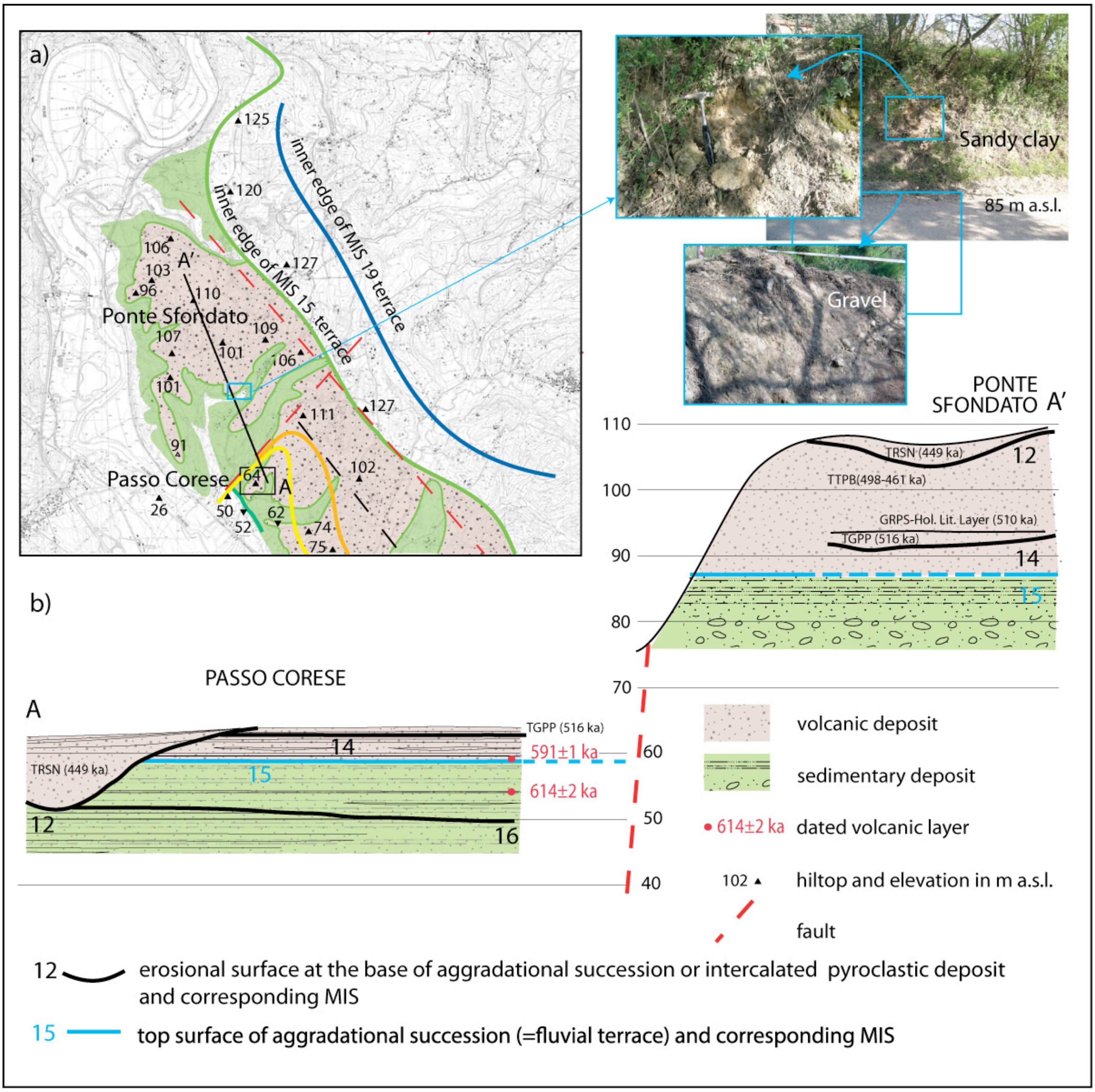

Figure S2-3 - a) Geologic map of the Ponte Sfondato area from field survey performed for the present study. Hilltop elevations and inner edges of the fluvial terraces reconstructed in this work are reported. 1:10.000 topographic base by Regione Lazio (http://dati.lazio.it/catalog/it/dataset/carta-tecnica-regionale-1991), available under Creative Commons Attribution License (https://creativecommons.org/licens/by/4.0/). Photos by authors.

b) Cross-sections showing stratigraphic setting in Ponte Sfondato and Passo Corese. Legend: TGPP: Tufo Giallo di Prima Porta, GRPS: Grottarossa Pyroclastic Sequence, TTPB: Tufi Terrosi con Pomici Bianche, TRSN: Tufo Rosso a Scorie Nere.

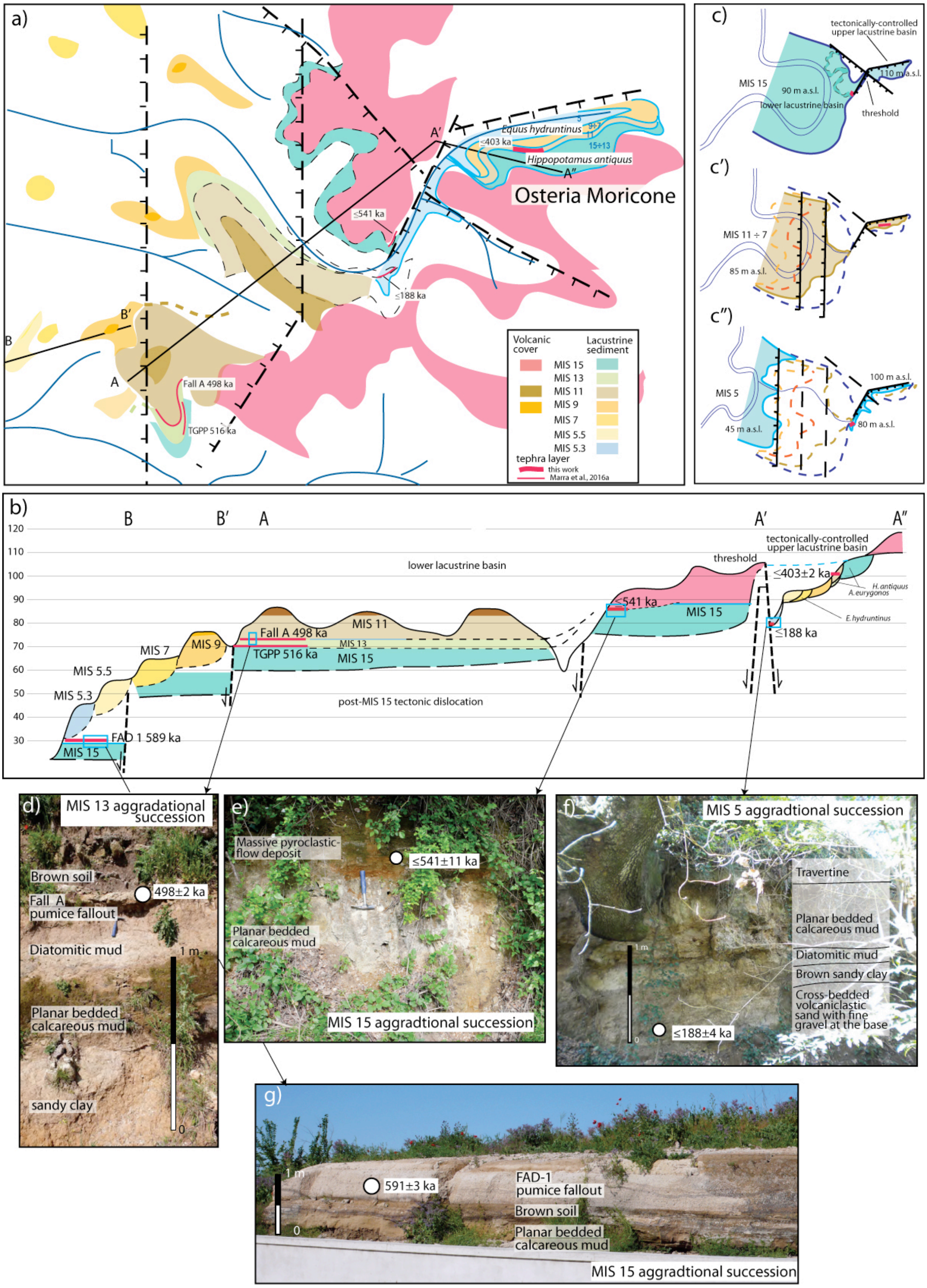

Figure S2-4 - a) Simplified geologic-geomorphologic map and cross-section (b) of the Cretone Basin (modified from [7] showing the geochronologic constrains on the sedimentary deposits of the lacustrine successions, and the inferred faults dislocating them, c-c") Structural evolution of the lacustrine basin, d-g) photographs of the sedimentary deposits and interbedded volcanic units providing correlation with the MISs (see [7] for detailed description). Photos by authors.

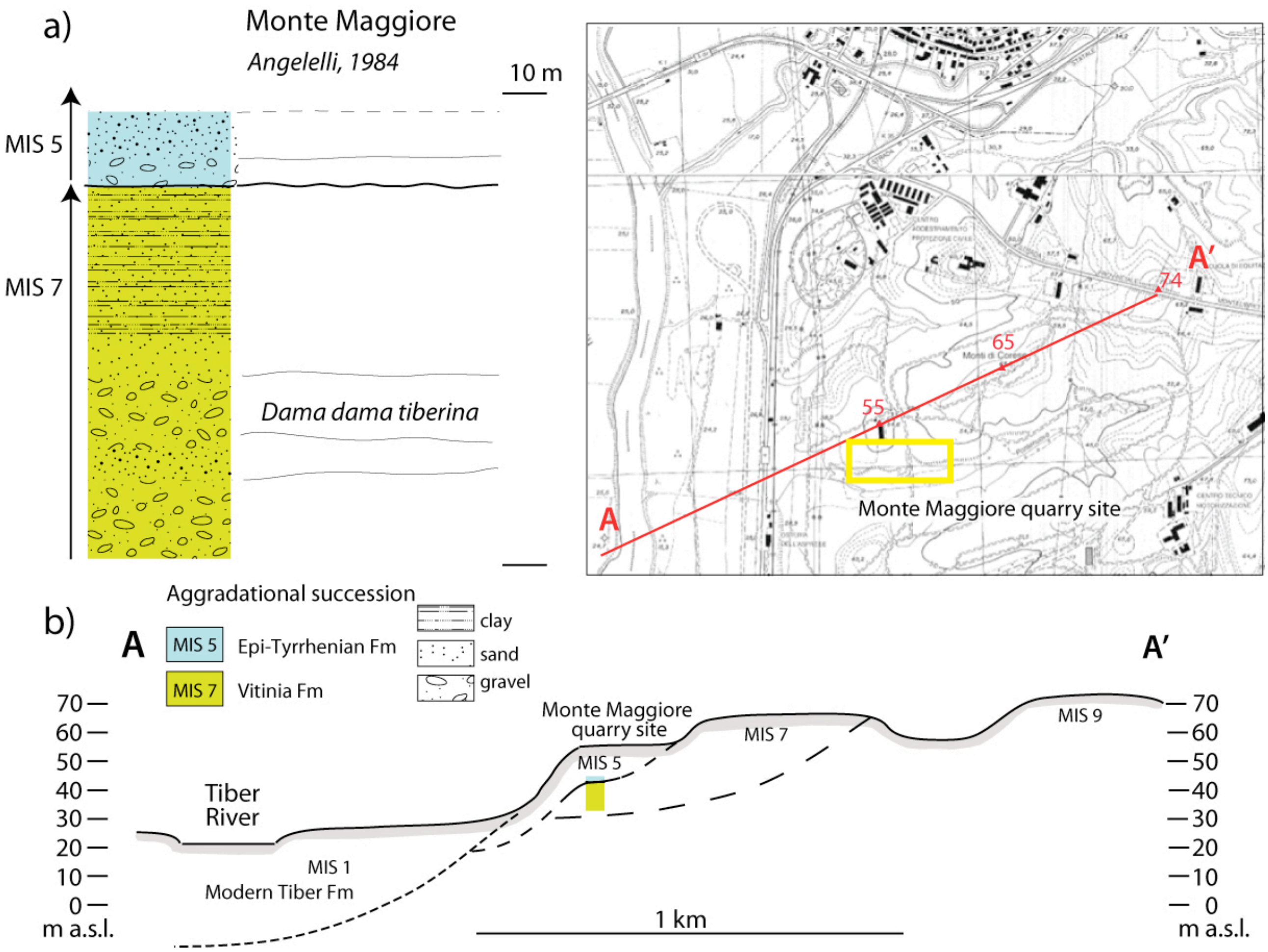

Figure S2-5 - a) Stratigraphic sketch of Monte Maggiore quarry and cross-section (b) drawn for the present paper showing the correlation with the MISs based on the occurrence of *Dama dama tiberina* remains [9]. 1:10.000 topographic base by Regione Lazio (http://dati.lazio.it/catalog/it/dataset/carta-tecnica-regionale-1991), available under Creative Commons Attribution License (https://creativecommons.org/licens/by/4.0/).

Geomorphological analysis

The statistic distribution of hilltops elevations has been assessed with a slightly different procedure with respect to that used by [8]. Figure 4b shows the recurrence of the elevations of all the topographic culminations detected on the 1:25.000 topographic maps. The peaks of elevation values are recognized assuming a mobile window of 7 m as a function of the data clustering. Classes of elevation ranges in Suppl. Figure #2-6 are analyzed for the total area and for the three geographic sectors A, B, and C, in order to outline possible gradient effects along the fluvial valley. These elevation ranges define the corresponding class of elevation pertaining to each paleo-surface (shaded vertical boxes in Figure 4b). A different color has been assigned to each class of elevation and used to distinguish the topographic points (triangles) of the corresponding paleo-surface in the Figure 4 legend. Slightly larger elevation ranges are used in some instances to account for all the topographic points. Open triangles indicate hilltops whose elevation is not statistically significant and are not associated with a paleo-surface. These elevations are interpreted to reflect topographic culminations representing eroded higher rank paleo-surfaces; for this reason, the same color used for the corresponding pristine paleo-surface is used also for these triangles.

A comparison of the results obtained here with those from the previous analysis [8] shows an overall match, with the following exceptions: i) two lowest classes of elevation values ranging 41-47 and 50-56 m a.s.l. are recognized in this work, instead of a single class ranging 46-50 m; ii) three peaks of elevation ranges, with a more statistically significant class at 151-157 m are recognized, rather than two at 145-150 m and at 160-165 m. Consequently, the correlation of the paleo-surfaces with the MISs has been slightly modified with respect to [8], in light of the new age constraints and detailed elevation assessment reported above.

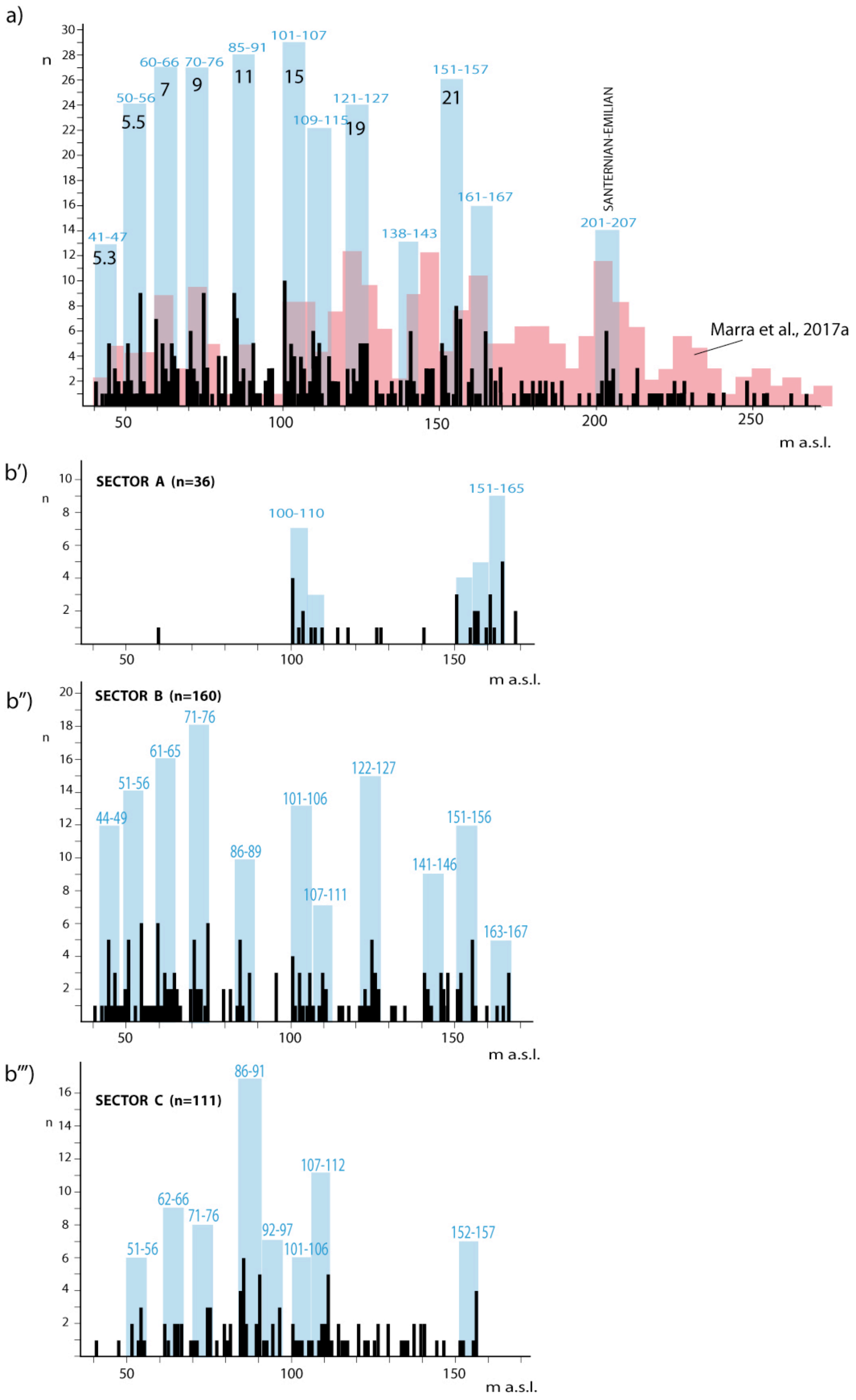

Figure S2-6 - a) Histograms reporting distribution and peaks of concentration for the topographic culminations for the total area, and b) for three different geographic sectors (A, B, C, see Figure 3 in the main text). A series of classes of elevation (vertical blue boxes), identifying the same number of paleo-surfaces are recognized and correlated with the sea-level highstands of marine isotopic stages (MISs), following criteria described in the text. Comparison with previous statistical assessment in a similar area by [8] is provided (red vertical boxes in a).

REFERENCES

1) Karner, D. B., Marra, F. & Renne P. R. The history of the Monti Sabatini and Alban Hills volcanoes: groundwork for assessing volcanic-tectonic hazards for Rome. *J. Volcanol. Geotherm. Res.* **107**, 185-219 (2001).

2) Marra, F., *et al*. Major explosive activity in the Sabatini Volcanic District (central Italy) over the 800-390 ka interval: geochronological - geochemical overview and tephrostratigraphic implications, *Quaternary Science Reviews* **94**, 74-101 (2014).

3) Marra, F., Jicha, B., & Florindo, F., 40Ar/39Ar dating of Glacial Termination VI: constraints to the duration of Marine Isotopic Stage 13. *Scientific Reports* **7**, 8908. DOI:10.1038/s41598-017-08614-6 (2017).

4) Karner, D. B. & Marra, F. Correlation of Fluviodeltaic Aggradational Sections with Glacial Climate History: A Revision of the Classical Pleistocene Stratigraphy of Rome. *Geol. Soc. Am. Bull.* **110**, 748-758 (1998).

5) Marra, F., Florindo, F., & Boschi, E. The history of glacial terminations from the Tiber River (Rome): insights to glacial forcing mechanisms. *Paleoceanography* **23**, PA2205 (2008). DOI:10.1029/2007PA001543.

6) Sottili, G. *et al*. Geochronology of the most recent activity in the Sabatini Volcanic District, Roman Province, central Italy. *Journal of Volcanology and Geothermal Research* **196**, 20-30 (2010).

7) Marra, F., et al. Chronostratigraphic constraints on Middle Pleistocene faunal assemblages and Acheulian industries from the Cretone lacustrine basin, central Italy. *Journal of Quaternary Science* **31**(7), 641–658, DOI: 10.1002/jqs.2889 (2016).

8) Marra, F., Florindo, F. & Petronio, C. Quaternary fluvial terraces of the Tiber Valley: geochronologic and geometric constraints on the back-arc magmatism-related uplift in central Italy. *Journal Scientific Reports* **7**, *2517,* DOI:10.1038/s41598-017-02437-1 (2017).

9) Angelelli, F. Descrizione e studio di resti di mammiferi del Plaistocene medio di Fara Sabina (Rieti-Lazio) conservati nelle collezioni del Servizio Geologico d'Italia. *Boll. Soc. geol. It.* **104**, 3-34 (1983-84).

10) Marra F. *et al*. A review of the geologic sections and the faunal assemblages of Aurelian Mammal Age of Latium (Italy) in the light of a new chronostratigraphic framework. *Quaternary Science Reviews* **181**, 173-199, doi.org/10.1016/j.quascirev.2017.12.007 (2018).

11) Di Stefano, G. & Petronio, C. Origin and evolution of the European fallow deer (*Dama*, Pleistocene). *Neues Jahrbuch für Geologie und Paläontologie Abhandlungen* **203**, 57-75 (1997).
