# Supplementary material for: Volcano-tectonic deformation in the Monti Sabatini Volcanic District at the gates of Rome (central Italy): evidence from new geochronologic constraints on the Tiber River MIS 5 terraces
Source: Sci Rep. 2019 Aug 8;9:11496. doi: 10.1038/s41598-019-47585-8 (PMC6687886; doi:10.1038/s41598-019-47585-8)
Supplement: Supplementary file 3 — Supplementarty Material 3 [file 41598_2019_47585_MOESM3_ESM.pdf]

# Volcano-tectonic deformation in the Monti Sabatini Volcanic District at the gates of Rome (central Italy): evidence from new geochronologic constraints on the Tiber River MIS 5 terraces

**Marra, F.[1]*, Florindo, F.[1], Jicha, B.[2], Nomade, S.[3], Palladino, D.M.[4], Pereira, A.[5,6], Sottili, G.[4], Tolomei, C.[1]**

1) Istituto Nazionale di Geofisica e Vulcanologia, Via di Vigna Murata 605, 00143 Rome, Italy
2) Department of Geoscience, University of Wisconsin-Madison, USA
3) Laboratoire des Sciences du Climat et de l'Environnement, LSCE/IPSL, CEA-CNRS-UVSQ, Université Paris-Saclay, F-91191 Gif-sur-Yvette, France
4) Dipartimento di Scienze della Terra, "Sapienza" Università di Roma, Piazzale Aldo Moro 5, 00185 Roma, Italy
5) UMR 7194 HNHP MNHN-CNRS-UPVD, Départ. Homme et Environ. du MNHN,1 rue René Panhard, 75013
6) Ecole française de Rome, Piazza Farnese, IT-00186, Roma, Italy

*Corresponding author: fabrizio.marra@ingv.it

## Supplementary Material #3 - Eruptive histories

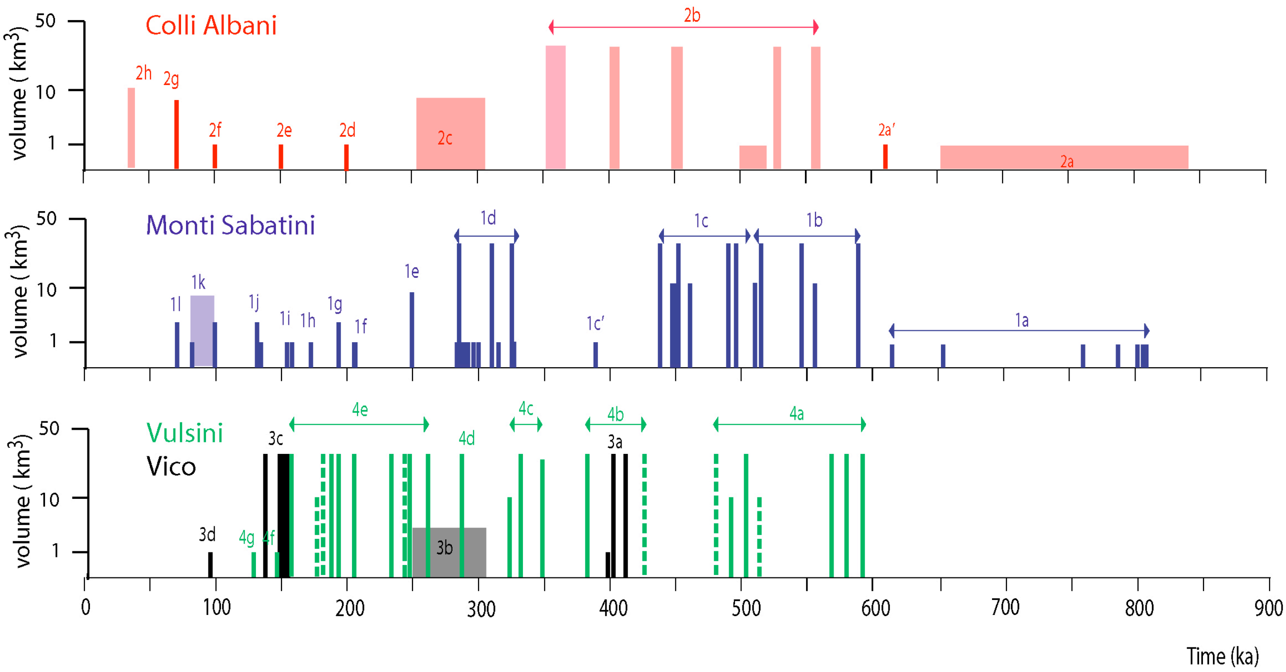


Eruptive history of the volcanic districts of the Roman Province reconstructed in this work from literature data, as follows. Vulsini (4a: Paleovulsini, 4b: Vulsini Fields, 4c: Bolsena phase, 4d: Case Pisello, 4e: Latera Phase, 4f: Monte Becco scoria cone, 4g: Bisentina Island; [1], and references therein); Vico (3a: 1st period, 3b: 2nd period, 3c: 3rd period, 3d: Monte Venere lavas, [2], and references therein); Monti Sabatini (1a: Paleo-activity, 1b: Morlupo activity, 1c: Southern Sabatini activity, 1c': Sant'Abbondio Ashfall Succession; 1d: Bracciano and Sacrofano activity, 1e: Pizzo di Prato eruption cycle, 1f: Monte Maggiore, Casale Francalancia scoria cones, 1g: Vigna di Valle eruption cycle, 1h: S. Bernardino maar, 1i: Lagusiello maar and Cornacchia lava, 1j: Prato Fontana lava, Baccano lower unit, 1k: Monte Broccoleto scoria cone, Stracciacappe, Le Cese, Piana dei Falliti, Acquarello maar, Baccano main and upper activity, 1l: Martignano maar, This work, [3, 4], and references therein); Colli Albani (2a: Paleo-activity, 2a': fosso Clleraso eruption unit, 2b Tuscolano-Artemisio phase, 2c: Monte delle Faete phase, 2d: Ariccia crater, 2e: Nemi crater, 2f: Valle Marciana crater, 2g: Albano 1st cycle, 2i: Albano 2nd cycle, [5, 6, 7, 8, 9, 10, 11] and references therein).

In ordinates are reported approximate values of erupted volumes in logarithmic scale; boxes indicate eruptive phases, vertical bars are single eruption cycles, dashed bars are known stratigraphic units units lacking precise geochronologic contraints.

REFERENCES

1) Palladino, D, Simei, S., Sottili, G., and Trigila, R., 2010, Integrated approach for the reconstruction of stratigraphy and geology of Quaternary volcanic terrains: An application to the Vulsini Volcanoes (central Italy), in Stratigraphy and Geology of Volcanic Areas (eds. G. Groppelli, and L. Viereck-Goette), The Geological Society of America Special Paper, 464, 63-84. doi: 10.1130/2010.2464(04).

2) Perini, G., Francalanci, L., Davidson, J.P., and Conticelli, S., 2004, Evolution and genesis of magmas from Vico volcano, Central Italy: multiple differentiation pathways and variable parental magmas, Journal of Petrology, 45, 139–182.

3) Sottili, G., Palladino, D.M., Marra, F., Jicha, B., Karner, D.B., Renne, P. (2010) - Geochronology of the most recent activity in the Sabatini Volcanic District, Roman Province, central Italy, Journ. of Volc. and Geoth. Res.. DOI:10.1016/j.jvolgeores.2010.07.003

4) Marra, F., Sottili, G., Gaeta, M., Giaccio, B., Jicha, B., Masotta, M., Palladino, D.M., Deocampo, D, 2014. Major explosive activity in the Sabatini Volcanic District (central Italy) over the 800-390 ka interval: geochronological - geochemical overview and tephrostratigraphic implications, Quaternary Science Reviews 94, 74-101.

5) Marra, F., Freda, C., Scarlato, P., Taddeucci, J., Karner, D.B., Renne, P., Gaeta, M., Palladino D.M., Trigila, R., Cavarretta, G., 2003. Post-caldera activity in the Alban Hills volcanic district (Italy): $^{40}Ar/^{39}Ar$ geochronology and insights into magma evolution. Bulletin of Volcanology 65, 227-247.

6) Marra F., D.B. Karner, C. Freda, M. Gaeta, P.R. Renne (2009) - Large mafic eruptions at the Alban Hills Volcanic District (Central Italy): chronostratigraphy, petrography and eruptive behavior, Journ. of Volc. and Geoth. Res., 179, 217-232 doi:10.1016/j.jvolgeores.2008.11.009

7) Marra, F., Gaeta, M., Giaccio, B., Jicha, B., Palladino, D., Polcari, M., Sottili, G., Taddeucci, J., Florindo, F., Stramondo, S., 2016. Assessing the volcanic hazard for Rome: $^{40}Ar/^{39}Ar$ and In-SAR constraints on the most recent eruptive activity and present-day uplift at Colli Albani Volcanic District, GRL 43, 6898-6906. doi:10.1002/2016GL069518

8) Freda, C., Gaeta, M., Karner, D.B., Marra, F., Renne, P.R., Taddeucci, J., Scarlato, P., Christensen, J.N., Dallai, L., 2006. Eruptive history and petrologic evolution of the Albano multiple Maar (Alban Hills, Central Italy). Bulletin of Volcanology 68, 567-591.

9) Giaccio, B., Marra, F., Hajdas, I., Karner D.B., Renne, P.R., Sposato A., 2009. $^{40}Ar/^{39}Ar$ and $^{14}C$ geochronology of the Albano maar deposits: implications for defining the age and eruptive style of the most recent explosive activity at the Alban Hills Volcanic District, Italy. Journal of Volcanology and Geothermal Research 185, 203-213.

10) Gaeta, M., Freda, C., Marra, F., Di Rocco, T., Gozzi, F., Arienzo, I., Giaccio, B., Scarlato, P., 2011. Petrology of the most recent ultrapotassic magmas from the Roman Province (Central Italy). Lithos 127, 298-308.

11) Gaeta, M., Marra, F., Freda, C., Arienzo, I., Gozzi, F., Jicha, B., Di Rocco,T., 2015. Paleozoic metasomatism at the origin of Mediterranean ultrapotassic magmas: constraints inferred from time-dependent geochemistry of volcanic products from Colli Albani (Central Italy), Lithos, 244, 151-164. http://dx.doi.org/10.1016/j.lithos.2015.11.034
